# Does the visual word form area split in bilingual readers? A millimeter-scale 7-T fMRI study

**DOI:** 10.1126/sciadv.adf6140

**Published:** 2023-04-05

**Authors:** Minye Zhan, Christophe Pallier, Aakash Agrawal, Stanislas Dehaene, Laurent Cohen

**Affiliations:** ^1^ Cognitive Neuroimaging Unit, INSERM, CEA, CNRS, Université Paris-Saclay, NeuroSpin Center, 91191 Gif/Yvette, France.; ^2^ Collège de France, Université Paris-Sciences-Lettres (PSL), 11 Place Marcelin Berthelot, 75005 Paris, France.; ^3^ Inserm U 1127, CNRS UMR 7225, Sorbonne Université, Institut du Cerveau, ICM, Paris, France.; ^4^ AP-HP, Hôpital de la Pitié Salpêtrière, Fédération de Neurologie, Paris, France.

## Abstract

In expert readers, a brain region known as the visual word form area (VWFA) is highly sensitive to written words, exhibiting a posterior-to-anterior gradient of increasing sensitivity to orthographic stimuli whose statistics match those of real words. Using high-resolution 7-tesla functional magnetic resonance imaging (fMRI), we ask whether, in bilingual readers, distinct cortical patches specialize for different languages. In 21 English-French bilinguals, unsmoothed 1.2-millimeters fMRI revealed that the VWFA is actually composed of several small cortical patches highly selective for reading, with a posterior-to-anterior word-similarity gradient, but with near-complete overlap between the two languages. In 10 English-Chinese bilinguals, however, while most word-specific patches exhibited similar reading specificity and word-similarity gradients for reading in Chinese and English, additional patches responded specifically to Chinese writing and, unexpectedly, to faces. Our results show that the acquisition of multiple writing systems can indeed tune the visual cortex differently in bilinguals, sometimes leading to the emergence of cortical patches specialized for a single language.

## Teaser

Bilingual English-Chinese but not English-French readers have language-specific cortical patches for written word recognition.

## INTRODUCTION

Half of humanity speaks more than one language, and many adults can read more than one language and master multiple writing systems. How does the visual cortex accommodate the recognition of written words in two languages, possibly using two different scripts? Much of previous research has shed light on the mechanisms of reading acquisition in a single script. Within the left ventral occipitotemporal cortex (VOTC), the recognition of written words mobilizes a small cortical area that has been termed the visual word form area (VWFA) (*1*). This region emerges during reading acquisition (*2*–*4*) and becomes tuned only to the script that the person has learned to read (*5*, *6*). In readers of all languages, the VWFA occupies a reproducible location within a mosaic of cortical regions specialized for the recognition of various categories of visual stimuli such as faces, bodies, objects, or places. This reproducible specialization is thought to be based on a combination of factors including foveal bias (*7*), preference for geometrical features (*8*), and preexisting connectivity to distant language areas (*9*, *10*). Longitudinal studies of schoolchildren show that the VWFA acquires its specialization for written words within the first few months of schooling (*3*). Its lesion, in literate individuals, results in pure alexia, a selective reading impairment (*11*).

How populations of neurons in the VWFA encode written words is not known [for proposals, see (*12*, *13*)], but one of its key macroscopic features is a sensitivity to the statistics of reading: The response of the VWFA increases as letter strings increasingly respect the statistical distribution of letters in real words, with an increasing gradient of sensitivity along the posterior-to-anterior axis along the VOTC (*14*–*16*). Thus, it is plausible that, during reading acquisition, neurons in the VWFA internalize the statistics of letters and their combinations. However, because of the limited spatial resolution of imaging methods, which frequently smooth and average data across many individuals, it is not known whether the macroscopic gradient along the VOTC results from a continuous increase in sensitivity to overall word similarity or from a chain of discrete cortical patches, each possibly responsive to a hierarchically higher-level orthographic component such as letters, bigrams, and larger chunks of letters (*14*), in part through interactive bottom-up and top-down influences (*16*).

Here, we ask how plasticity allows this architecture to adapt to reading in two different languages in bilingual readers. Do distinct cortical patches implement reading in different languages? The statistical learning hypothesis above leads to contrasting predictions depending on whether the two languages use the same alphabet (e.g., English and French) or two very different scripts, typically alphabetic versus logographic (e.g., Chinese). When the two languages use the same alphabet, words share similar visual features in both languages, and the visual cortex projects to the same distant language areas (*17*). One may therefore predict that the same cortical patches would be used in both languages. Local patches of visual cortex would compile letter statistics without any consideration of which language they transcribe and should therefore be sensitive to the overall orthographic statistics (*18*), pooled across both languages (in proportions that may vary depending on the dominance of one language over the other for a given individual). There is currently no imaging evidence for language-specific patches in the VOTC, either between monolingual individuals (*19*) or within bilingual individuals (*20*). Accordingly, we know of no reports of developmental dyslexia or pure alexia differently affecting two languages using the same alphabet (*21*). Nevertheless, it remains possible that distinct cortical patches or columns are specialized for one or the other language and incorporate the orthographic statistics of only one language. This fine-grained specialization may have escaped the relatively coarse spatial resolution of previous 3-T functional magnetic resonance imaging (fMRI) studies, especially using group-level averaging, and the even coarser grain of brain lesions.

Conversely, in bilingual readers of an alphabetic and a logographic script, written words differ in the number of learned characters, visual complexity, component shapes, and overall contour area. These factors make it more likely that the VOTC should develop script-specific cortical patches (*22*), with each patch encoding the orthographic regularities of a single language. Nevertheless, statistical analyses suggest that all scripts rely on similar statistics of line junctions (*23*). Thus, it may still be useful for the visual system to share the same cortical resources between two distinct reading systems. Existing brain imaging data, however limited, mostly support this second possibility. Neither between monolinguals (*19*) nor within bilinguals (*17*) is there any strong evidence for distinct script-selective regions. However, multivariate techniques can successfully decode Chinese from English words in the left VOTC (*17*), which may point to specialization beyond the usual resolution of fMRI. In Japanese readers who are proficient in both the syllabic Kana and the logographic Kanji scripts, a double dissociation has been reported between two types of pure alexic patients, with a predominant deficit for either Kana or Kanji (*24*). In developmental dyslexia, a single dissociation between English (impaired) and Japanese (preserved) has been reported (*25*). Thus, the existence of script-specific cortical patches is a plausible hypothesis, whose empirical assessment requires high-resolution individual brain imaging.

Here, we study the organization of visual word recognition in the VOTC of bilingual participants, while maintaining high spatial precision using individual analyses of high-resolution 7-T fMRI (1.2-mm isotropic voxels). We first imaged 21 bilingual readers of English and French, two languages written with the same alphabet but different orthographic statistics, and then 10 bilingual readers of English and Chinese, two languages based on alphabetic and logographic scripts with very different visual features. In each case, following up on our previous work (*15*), we created a hierarchy of stimuli with increasing similarity to real words. This design allowed us to assess the cortical implementation of orthographic statistics in three different languages, to look for cortical patches specialized for orthographic components of alphabetic versus logographic scripts, to assess the existence of specialization for one of two available languages, whether they share the same script, and to study the modulation of these findings by language dominance.

## RESULTS

### Experiment 1: Reading in English-French bilinguals

#### Localizer for visual categories and overall reading circuit

**Fig. 1.**
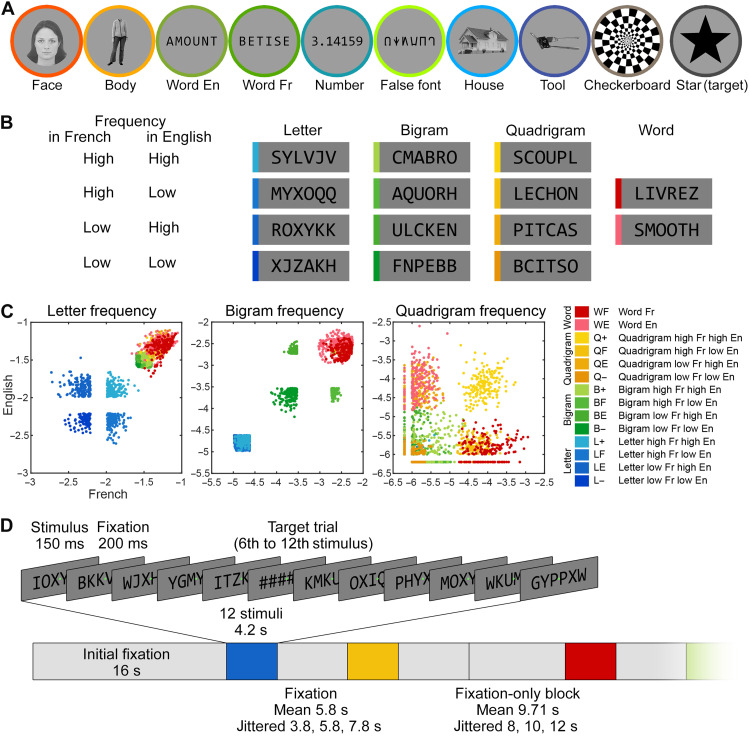
Stimuli and procedure for Experiment 1 with English-French bilingual readers. (**A**) Examples of the nine categories of visual stimuli and the target used in the localizer. (**B**) Examples of the 14 categories of visual stimuli used in the main fMRI runs, consisting of six-letter strings whose similarity to words was systematically manipulated. The frequency of letters, bigrams, and quadrigrams, as well as the effect of lexicality, were manipulated orthogonally in English and French, resulting in 12 types of nonword letter strings, as well as English and French words. (**C**) The frequency of letters, bigrams, and quadrigrams in English (*y* axis) and in French (*x* axis) for all 14 categories of stimuli (2520 stimuli in total; one dot indicates one stimulus; unit = log_10_ counts per million). The English and French real words (WE and WF) were matched to the corresponding high-frequency quadrigram stimuli (QE and QF) in all three parameters. (**D**) Organization of an fMRI run, consisting of an alternation of fixation periods and homogeneous blocks of fast stimulus presentation.

We recruited 21 English-French bilingual readers (seven dominant in English, seven fully bilingual, and seven dominant in French). Participants were first imaged using a localizer for various visual categories, including words in both languages. They passively viewed blocks of English or French words, Arabic numbers, false font strings, faces, bodies, houses, tools, and checkerboards, while they were asked to detect an occasional star to keep their attention focused (Fig. 1A).

**Fig. 2.**
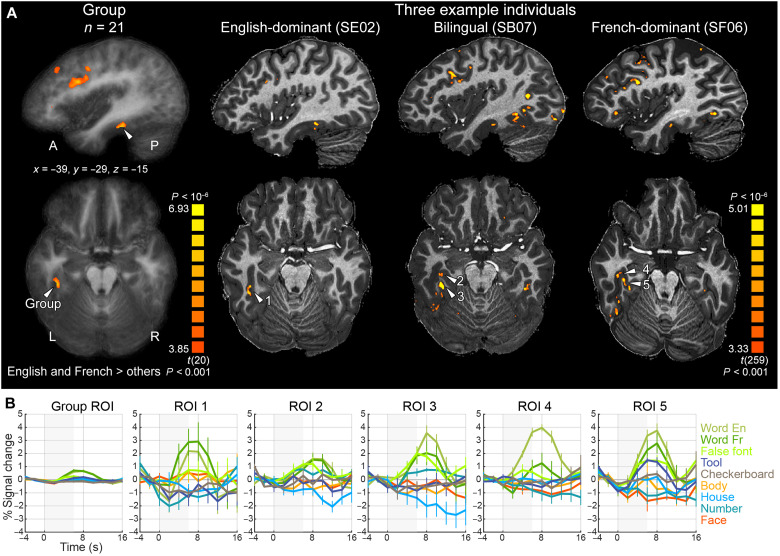
Activation to written words in individual English-French bilingual readers, illustrating the need for single-subject analyses. (**A**) In VOTC, the group-level contrast of words > faces, bodies, houses, and tools showed only one anterior word-specific cluster (TAL *Y* = −29, column 1). However, each individual participant actually showed robust word-specific cortical clusters/patches around the usual location of the VWFA, as shown in one participant per language group (columns 2 to 4; see fig. S2 for activation for all 21 participants). Overlap between participants was reduced because of individual variability and the small size of these clusters/patches. (**B**) Averaged time courses for each category of stimuli in the clusters marked with white arrowheads in (A). The shaded area represents stimulus block duration. Error bars denote SEM. Note that the apparent higher activity in ROI 4 for English than French words (*P* = 0.0063) did not survive whole-brain cluster thresholding (*P* < 0.001) and was not replicated in the main fMRI runs (*P* = 0.434).

We first examined whole-brain word-specific activation at the group level. To facilitate intersubject averaging, the data were smoothed with a Gaussian filter with full width at half maximum of 6 mm. We examined the contrast of English and French words > faces, bodies, houses, and tools, with a cluster size threshold corrected for multiple comparisons using Monte-Carlo simulations (*P* < 0.001, α < 0.05, resulting cluster size > 74 functional voxels, 1.2 mm isotropic). We found several word-specific clusters along the bilateral superior temporal sulci (STS) and inferior frontal gyri (IFG), with left predominance. In the left VOTC, we did not find the usual VWFA [around Talaraich (TAL) coordinate *Y* = −56] but found a more anterior cluster in the left occipitotemporal sulcus (OTS), peaking at *Y* = −29 (Fig. 2A). Group-level comparisons of English and French words revealed only two small clusters in the right cuneus and ventromedial prefrontal cortex (α < 0.05, cluster size > 27 functional voxels) that were more activated by English than by French words. The opposite contrast showed no significant activation.

As described in a previous study (*26*), the absence of a VWFA cluster in the group analysis likely resulted from individual anatomical and functional variability. Compared to 3-T data, 7-T images have enhanced white-gray matter contrast, and the activated clusters/patches are smaller (diameters were typically within 10 mm), largely confined to the gray matter, and with little overlap between participants. Thus, classical brain-wide group analyses are largely inoperative. In contrast, in every single participant, without data smoothing, we observed focal word-specific clusters along the gray matter of the inferior occipital sulcus (IOS) and OTS in the vicinity of the VWFA, showing robust activation time courses (*P* < 0.001 uncorrected; Fig. 2 and fig. S2). The size of these clusters changed only minimally when using a more stringent voxelwise threshold of *P* < 0.0005. Therefore, all of our subsequent analyses were based on the signals extracted from 773 single-subject word-specific cortical patches, followed by group-level statistical inferences.

In 17 of 21 participants, word-specific clusters in the VOTC were bilateral, and the remaining 4 participants showed only left-hemisphere clusters. The mean number of activated clusters and voxels was much larger in the left than in the right hemisphere (paired *t* tests; mean of 10.8 versus 3.5 clusters per participant, *P* = 7.50 × 10^−10^; mean of 853 versus 192 voxels, *P* = 4.58 × 10^−7^). Around the fusiform gyrus, clusters were mostly dispersed along the IOS and OTS (fig. S2) and did not follow any obvious grouping pattern. Again, there were more clusters around the left than the right fusiform region (paired *t* test; mean of 6.4 versus 1.1 clusters per participant, *P* = 4.88 × 10^−10^; mean of 433 versus 42 voxels, *P* = 9.88 × 10^−7^).

Previous research has emphasized the similarities between word and face recognition [e.g., (*7*, *27*)]. Faces also require foveal processing and elicit category-specific activation in the VOTC, mesial and adjacent to word-specific activations, but with right-hemispheric predominance (*28*). In parallel to what we did for words, we identified face-specific clusters in the VOTC (contrast: faces > English and French words, bodies, houses, and tools; *P* < 0.001 uncorrected; fig. S2). We found bilateral VOTC clusters in all participants, more in the right than in the left hemisphere (paired *t* tests; 6 versus 4.05 clusters, *P* = 0.03; 368 versus 171 voxels, *P* = 4.14 × 10^−5^). Their location roughly followed a posterior-to-anterior clustering pattern that was reproducible across participants, corresponding to the previously described occipital face area, fusiform face area, and anterior face patch (*29*). Some face-specific clusters were adjacent to word-specific clusters, e.g., on opposite banks of the same sulcus (see, e.g., SB03 and SF05 in fig. S2), and, occasionally, they overlapped by a few voxels.

For both word- and face-specific clusters, we observed anterior activation in the fusiform and OTS regions (TAL *Y* coordinates, −50 to −22). These anterior clusters are often absent in fMRI studies of the VWFA but have been reported in intracranial studies [e.g., (*30*, *31*)]. We consistently found them here because, early in the experiment, we optimized the placement of the acquisition slices and improved the signal-to-noise ratio (SNR) affected by the signal dropout around the ear canals (*32*). This signal dropout was visible in the very first three participants (fig. S1), although the artifact was anterior and did not affect the VWFA. The SNR around the ear canals was considerably improved in all subsequent participants.

We also used the localizer to search for significant activation differences between English and French words. We did not find any consistent language-specific activation (within-subject threshold, *P* < 0.001 uncorrected). Only six participants show some putative language-specific clusters, all outside the VOTC, distributed in left and right supramarginal gyri, STS, intraparietal sulcus (IPS), and lateral frontal areas [inferior frontal sulci (IFS)/IFG]. However, the language preference of these clusters was not reproduced in the main fMRI runs described below and was, therefore, not analyzed further.

Because of the lack of language-specific clusters, we used the bilingual word-specific contrast (English and French words > faces, bodies, houses, and tools) to define regions of interest (ROIs) for subsequent analyses. To further rule out the possibility that we may have missed language-specific clusters in this bilingual word-specific contrast, we also examined the voxels of two additional language-specific contrasts: English words > faces, bodies, houses, and tools; and French words > faces, bodies, houses, and tools (both *P* < 0.001 uncorrected, cluster size threshold > 4 voxels). The voxels in the language-specific contrasts mostly overlapped with those in the bilingual word-specific contrast (English: mean = 91.9% overlap with bilingual voxels, SD = 11.3%; French: mean = 86.1%, SD = 17.0%) and showed an even higher overlap within the VOTC (English: mean = 95.8%, SD = 5.2%; French: mean = 94.8%, SD = 7.3%). Supplementary analysis of the main fMRI runs failed to show reproducible language specificity again: Even when voxels were isolated on the basis of their apparent responsiveness to a single language in the localizer (e.g., English but not French), the language specificity for the two languages was not consistent across conditions and was not reproducible between the localizer and the main fMRI runs (see Supplementary Text and fig. S3).

#### A hierarchy of stimuli separately manipulating the statistics of English and French

In the main fMRI runs, participants passively viewed miniblocks of 12 consecutive six-letter strings presented at a fast rate (stimulus onset asynchrony = 350 ms; see Fig. 1, and Materials and Methods). Their task was to detect an occasional string of hashtags (######), which occurred randomly in 40% of the blocks. We used a design similar to Vinckier *et al.* (*15*) but adapted for bilinguals, by carefully generating pseudo-words that orthogonally varied the frequency of a given orthographic component in English and in French. For example, for the letter level, we generated stimuli whose average letter frequency was low in both languages, low in one language but high in the other, or high in both—resulting in a 2 × 2 design with low/high frequency in English × low/high frequency in French. By manipulating letters, bigrams, and quadrigrams in this way, we obtained 12 categories of pseudo-word stimuli increasingly similar to real words, crossing English/French × high/low frequency × letters/bigrams/quadrigrams, to which we further added two sets of real English and French words (see Fig. 1, B to D, and Materials and Methods). Each letter string was presented only once in the experiment.

We defined 773 bilingual word-specific ROIs in individual participants using the localizer data (*P* < 0.001 uncorrected, cluster size > 4). We verified that almost all of these ROIs also showed above-baseline activity for words (English + French) in the main fMRI runs (749 of 773 ROIs, including 294 of 299 VOTC ROIs). We then extracted the activity values (β, equivalent to % signal change) for the 14 conditions of the main fMRI runs and averaged them across voxels in each ROI.

#### A spatial gradient of word similarity

**Fig. 3.**
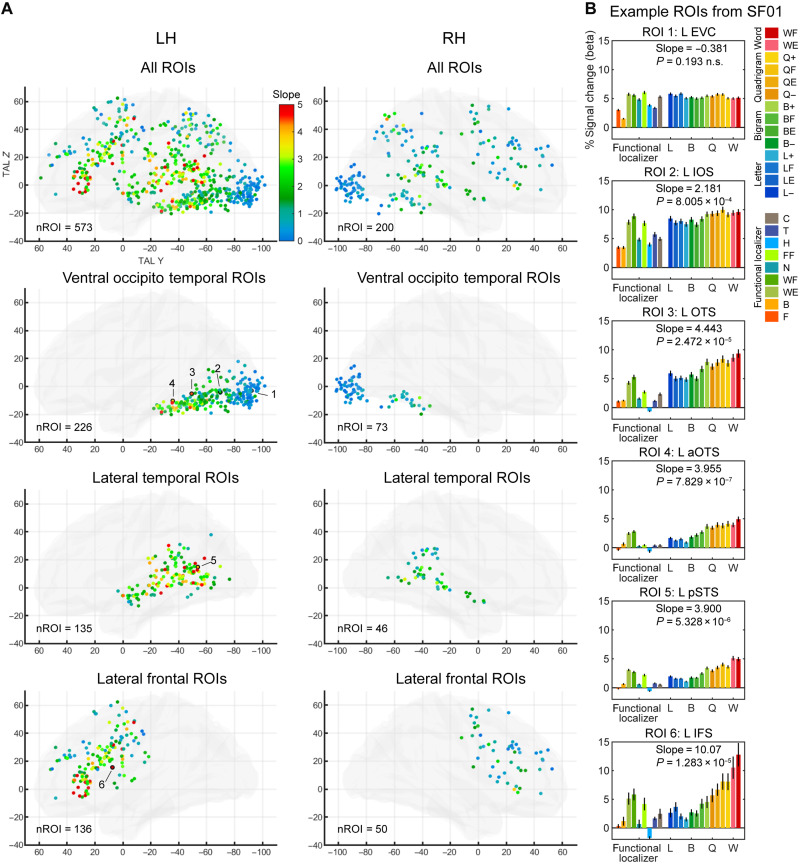
The word-similarity effect in English-French bilingual readers. (**A**) Word similarity was modeled as the slope of a linear regression over the 14 types of stimuli ranging from low-frequency letters to real words (see Materials and Methods for details). Transparent hemispheres (LH and RH) show the slope value (color-coded) in each of the 773 word-specific ROIs defined by the localizer. There is a posterior-to-anterior gradient of increasing word-similarity effect in the VOTC and two other putative gradients in the STS and the lateral frontal cortex. (**B**) Activity profile in six ROIs of a representative participant [SF01; see (A) for ROI localization]. Bars represent the activity of each condition in the localizer (left) and the main fMRI runs [right; see color legend at right; note that color codes in (A) and (B) are unrelated]. Error bars denote SEM across voxels within each ROI. aOTS, anterior OTS; pSTS, posterior superior temporal sulcus.

We first assessed the overall effect of stimulus similarity to real words, pooling across both languages without assuming language specificity, by assigning weights to the 14 conditions, ranging from 1 for strings with low-frequency letters in both French and English to 10 for real words. We then normalized these weights between 0 and 1 and used them to fit a linear regression to the activity per condition within each ROI (see Materials and Methods and Fig. 3). Whenever we use the term “slope,” we refer specifically to this regression coefficient, which quantifies the word-similarity effect. A significant slope in an ROI would indicate a word-similarity effect in that ROI.

Of the 773 word-specific ROIs identified across all participants, 64.5% (499 of 773) showed a significant slope [*P* < 0.05, false discovery rate (FDR) *q* < 0.05; Fig. 3A, top row]. ROIs were grouped into three broad anatomical regions (VOTC, lateral temporal, and lateral frontal; Fig. 3A, rows 2 to 4). Examining the bilateral VOTC, we observed a posterior-to-anterior increase in the slope of the word-similarity effect along the VOTC (Fig. 3, second row), similar to the spatial gradient observed in (*15*). We use the term “gradient” here to denote the gradual changes of a functional property across cortical space. In the left VOTC, the gradient of word similarity was highly reproducible across participants: The slope of the word-similarity effect was positively correlated with the TAL *Y* coordinate of the clusters in 19 of 21 participants [all individual *P* < 0.028; group-level *t* test of the regression coefficient against 0, mean coefficient = 0.0443, *t*(20) = 16.190, *P* = 5.84 × 10^−13^]. When the analysis was restricted to ROIs around the left fusiform gyrus, excluding the early visual cortex (EVC) ROIs, the gradient was still significant [VOTC ROIs anterior to the posterior collateral sulcus, mean coefficient = 0.0444, *t*(20) = 7.101, *P* = 6.98 × 10^−7^].

In the right VOTC, the gradient was also significant [mean coefficient = 0.0252, *t*(13) = 3.301, *P* = 0.0057], but the coefficients were smaller than in the left hemisphere [paired *t* test for participants with at least three ROIs in the right hemisphere, *t*(13) = 2.602, *P* = 0.022], indicating a left predominance.

While we focus here on the VOTC, we also note that there was a trend for a similar gradient in the lateral frontal ROIs around the IFG (Fig. 3), similar to what we previously observed at a lower resolution (*15*). We examined this putative gradient in more detail, by performing a principal components analysis (PCA) on the TAL *Y* and *Z* coordinates across ROIs and using the first PCA component as the coordinates of the ROIs along the main axis of the gradient. We then computed the correlation across ROIs between these coordinates and the word-similarity slope. Of the 11 of 21 participants who had at least three left lateral frontal ROIs, only 3 of 11 showed a significant correlation (all *P* < 0.015). On closer inspection, the ROIs with the steepest word-similarity slope (slope > 5; red ROIs in Fig. 3, bottom left) were tightly localized around the IFG, particularly in the pars triangularis and pars orbitalis, while only a few were located in the IFS. We concluded that the IFG did not harbor a spatial gradient like the VOTC but rather a focal sensitivity to word similarity.

The left VOTC gradient could also be confirmed by comparing words to control stimuli in the localizer. Activity evoked by false fonts and numbers decreased from posterior to anterior VOTC ROIs (negative regression coefficient values when fitting TAL *Y* coordinates to the activity, Fig. S4, A and B), but this decrease was much less pronounced for English and French words (mean regression coefficients: false fonts = −0.0425, numbers = −0.0484, French words = −0.0082, English words = −0.0111; paired *t* tests, all *P* < 2.4397 × 10^−10^). The difference between words and control stimuli became massive in anterior ROIs (TAL *Y* > −50).

We also computed a selectivity index for words versus other categories, separately for left and right ventral ROIs, as [word activity − other activity] / [word activity + other activity], where word activity was the average activity evoked by English and French words, and other activity was the average activity to faces, bodies, houses, and tools. The activity of all conditions was padded with the value of the condition with the most negative β value, so that all resulting β values were non-negative, and the resulting selectivity index would range between −1 and 1. In left ventral ROIs, the word selectivity index increased monotonically with the TAL *Y* coordinate (fig. S4C; mean regression coefficient = 0.005, one-sample *t* test against zero, *P* = 9.52 × 10^−10^) and continued to increase toward one anterior to the VWFA (TAL *Y* around −56). In summary, two independent analyses revealed a clear cortical gradient of increasing word selectivity from posterior to anterior VOTC.

#### Dissecting the word-similarity effect

Next, we examined in detail the properties of the word-similarity effect in the VOTC (*15*). First, we asked whether the word-similarity effect is an exclusive property of word-specific clusters or whether it reflects a general feature of the VOTC as a whole. We computed the word-similarity slope separately within word-specific and face-specific clusters (the latter while excluding voxels overlapping with the word-specific clusters). We found that while 51% of bilateral VOTC word-specific clusters showed a significant slope of the word-similarity effect (153 of 299 ROIs at FDR *q* < 0.05), this was the case for only 9.8% of face-specific clusters (26 of 264 ROIs at FDR *q* < 0.05). Most of the latter ROIs were located in the left hemisphere (23 of 26 ROIs) and were located around the OTS, IOS, posterior collateral sulcus, and mid-fusiform gyrus, and 12 of them had 1 to 14 overlapping voxels with word-specific clusters. This analysis indicates that sensitivity to word similarity exists almost exclusively within discrete word-specific cortical patches and their close vicinity, and is not a widespread feature of the entire VOTC: The broad, quasi-continuous extension of the word-similarity effect previously observed by Vinckier *et al.* (*15*) was due to lower resolution data, image smoothing, and intersubject averaging.

Second, we evaluated the hypothesis that a succession of patches along the posterior-to-anterior axis of the VOTC may be selectively sensitive to increasingly complex orthographic components. According to this hypothesis, patches would be specialized for increasingly larger components of words, from frequent letters to bigrams to quadrigrams, culminating in anterior regions sensitive to lexical status (*14*). Following this hypothesis, the word-similarity effect that we observed could actually be due to discrete steps of activity increase, with each patch responding to an orthographic unit of a certain grain size. Alternatively, the activity increase in the word-similarity effect could be truly continuous in nature: As stimuli become increasingly similar to words in the orthographic lexicon, they would generate a bottom-up activation that increases monotonically, or, perhaps, they could also receive progressively increasing top-down feedback signals from higher areas (*16*). To test these hypotheses, at this stage irrespective of language, we contrasted conditions in which the frequency of orthographic components was high versus low in both languages (L+ versus L−, B+ versus B−, and Q+ versus Q−) and contrasted words (WE and WF) versus pseudo-words (QE and QF) for the lexicality effect.

We first examined the prevalence of whole-brain activation across individual participants. Lexicality elicited relatively consistent differences between words and pseudo-words in 76% (16 of 21) of participants, with clusters of higher activation for words in 15 participants and clusters of higher activation for pseudo-words in 7 participants (*P* < 0.001 uncorrected, cluster size > 4). Higher activation for words (number of participants in parentheses) was mainly found in language areas along the STS (13), in IFS/IFG (8), precentral sulcus (5), supramarginal gyrus/planum temporale (5), middle frontal gyrus (MFG) (3), supplementary motor area (SMA) or preSMA (3), and IPS (3). Only four participants showed positive activation in the VOTC, and, in three of them, the activation was located around the middle occipitotemporal gyrus and superior to the VWFA. Conversely, higher activation for pseudo-words was found mainly along the brain midline and near the frontal pole, including the retrosplenial cortex (3), parieto-occipital junction (3), precuneus (2), medial superior frontal gyrus (2), and MFG (2). None of the other planned contrasts elicited any activation in >5 participants, and the consistency across participants was low. In summary, while lexicality effects (word versus pseudo-word) elicited discrete activity jumps, other levels (letters, bigrams, or quadrigrams) did not.

**Fig. 4.**
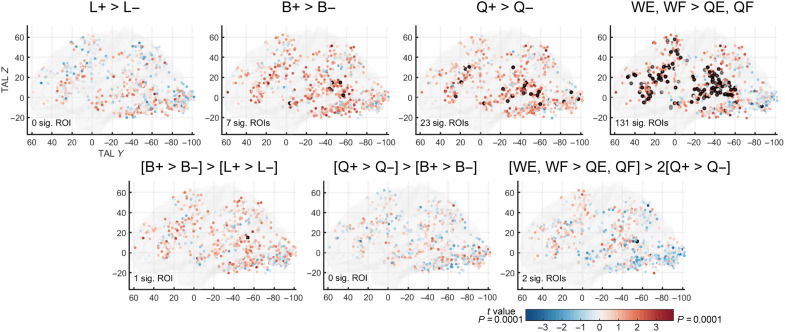
Contribution of letter, bigram, and quadrigram frequencies, as well as lexicality, to the overall word-similarity effect in English-French bilingual readers. Top row: Contrasts of high minus low frequency conditions for letters, bigrams, and quadrigrams and contrast of real words minus matched quadrigrams in the 773 word-specific ROIs. Bottom row: Pairwise comparisons between the contrasts of the top row. ROIs circled in black are significant after correction for multiple comparisons. (FDR *q* < 0.05, corrected within the VOTC, lateral temporal, and lateral frontal regions, respectively. No FDR correction for the 107 ROIs falling outside these three regions). ROIs in the VOTC showed no effect, providing no evidence that they are specialized for a specific type of orthographic component. Lateral temporal and frontal ROIs were mainly sensitive to lexical status and quadrigram frequency.

We therefore moved to analyses within the 773 word-specific ROIs obtained from the localizer analysis. For each ROI, we averaged time courses across voxels and recomputed the general linear model (GLM) and contrasts (ROI-GLM). We examined the same contrasts as in whole-brain analyses (Fig. 4, top row), as well as the pairwise differences in frequency and lexicality effects between successive component types (B versus L, Q versus B, and W versus Q; Fig. 4, bottom row). Overall, we found only a very small number of ROIs sensitive to frequency for each component level (L, none; B, 7; Q, 23), of which only a few were in the VOTC (L and B, none; Q, 7). The lexicality effect (W > Q) again showed more robust activation in lateral temporal and frontal areas (ROIs from nine participants, including six SB, two SF, and one SE) but, again, with few VOTC ROIs (four ROIs from two SB). This scarcity of significant ROIs is consistent with whole-brain analyses and indicates that reading-related patches are not selectively sensitive to discrete, specific levels of orthographic structure but are progressively activated by increasingly word-like stimuli. Consistent with this conclusion, note that the vast majority of ROIs already showed above-baseline activity even under the infrequent letter condition (593 of 773 ROIs across the brain and 285 of 299 VOTC ROIs at FDR *q* < 0.05). While it remains possible that successive word patches respond to other increasingly invariant features of reading such as size, case, or font invariance (*33*), as found in face recognition (*29*, *34*), their organization does not neatly separate according to the exclusive presence of letters, bigrams, or quadrigrams.

#### Do distinct cortical patches specialize for English versus French?

The main goal of our experiment was to search for putative cortical patches with a specific tuning to English or French. To this end, lexicality and frequency statistics were manipulated orthogonally between the two languages.

##### Differences between English and French real words

The localizer experiment showed no consistent differences between activations to English and French words. This conclusion was confirmed in the main fMRI runs, despite having much more data than in the localizer (15 versus 5 repetitions per condition). Only four participants showed seemingly language-specific activations in a total of 29 clusters, but these findings were not consistently replicated in the localizer: Within the 29 main experiment clusters, we tested the differences between English and French in the localizer data with ROI-GLM. The effect of language was nonsignificant (22 of 29 ROIs) or weak (0.005 < *P* < 0.05; 6 of 29 ROIs). Similarly, in the 773 word-specific ROIs, ROI-GLMs identified only six ROIs with a significant language difference (FDR *q* < 0.05).

##### Differences between English and French sublexical statistics

Within word-specific ROIs, for each sublexical component (letter, bigram, and quadrigram), we compared stimuli whose component frequency diverged between English and French (LE versus LF, BE versus BF, and QE versus QF). Only a single ROI showed a language difference for bigram frequency. We also assessed the full 2 × 2 design by probing (i) the main effect of frequency separately for each language (e.g., [LF, L+] versus [LE, L−] for the main effect of letter frequency in French) and (ii) the interaction term, which would indicate that the frequency effect was significantly larger in one language than in the other. Crucially, this interaction term was never significant for any of the sublexical components. We found only two ROIs with a significant main effect of bigram frequency in English and a few ROIs with a significant main effect of bigram or quadrigram frequency in French (34 and 12 ROIs, respectively). These ROIs came from seven participants (six SB and one SF), although the majority of ROIs actually came from one French participant (SF01; bigram effect, 20 ROIs; quadrigram effect: 5 ROIs).

##### Effects of individual language dominance

Our 21 participants consisted of seven English-dominant, seven French-dominant, and seven balanced bilinguals. We examined whether their language profiles were reflected in the brain, namely, the activity difference between English and French conditions across individual participants. For each participant, we computed a behavioral score of language dominance based on the number of words they read aloud in 1 min: ([English words − French words] / [English words + French words]). To obtain a comparable index at the brain level, we merged bilateral word-specific ROIs into bigger anatomical regions or subregions for each participant. The three broad anatomical regions were the same as defined before (VOTC, lateral temporal, and lateral frontal; see Fig. 3A). For a detailed examination of the VOTC and, in particular, around the fusiform area, we further examined subregions of ROIs surrounding the fusiform gyrus according to specific individualized anatomical locations, including the mid-fusiform sulcus (mFS) and the IOS-OTS subregions, and grouped all VOTC ROIs posterior to the fusiform region into an EVC region. Within each region, we computed the average activity difference evoked by English versus French conditions in the localizer and main runs (WE−WF, LE−LF, BE−BF, and QE−QF). Last, we correlated the activity difference with each participant’s behavioral language dominance score.

For activity differences between English and French words of the main fMRI runs, as expected, no significant correlation was found in the EVC [*r*(19) = −0.183, *P* = 0.427]. However, a negative correlation was found in the fusiform region [*r*(19) = −0.550, *P* = 0.0098, FDR-corrected for five comparisons within each region; fig. S5], indicating that words in the dominant language elicited lower activity, presumably due to lower effort. Dissecting the fusiform region, this negative correlation was found in the IOS-OTS subregion [*r*(19) = −0.661, *P* = 0.0011], but not in the mFS [*r*(15) = 0.357, *P* = 0.159]. Outside the fusiform region, a similar negative correlation was also found in lateral temporal and lateral frontal regions, although not surviving FDR correction [*r*(19) = −0.548 and *r*(14) = −0.600; uncorrected *P* = 0.0102 and *P* = 0.014, respectively]. None of the other sublexical components (LE−LF, BE−BF, and QE−QF) correlated with the behavioral language dominance in any of the regions/subregions (all *P* > 0.064). The language difference in the localizer did not show any significant correlation either (all *P* > 0.339).

#### Summary of the English-French experiment

With the higher resolution afforded by 7 T compared to previous 3-T studies, the VWFA became subdivided into a multitude of cortical patches that were highly word-specific and required single-subject analyses. Activity showed a robust word-similarity effect in 64.5% of word-specific ROIs and a posterior-to-anterior increase in this effect along the VOTC, reproducible in every single participant. However, all of these ROIs were coactivated by both English and French words, and we did not find consistent differences between languages, in either whole-brain or ROI analyses.

The lack of language differences in the VOTC of English-French participants may be due to the fact that English and French use identical alphabets. To examine whether widely different scripts lead to activity differences in the VOTC, we extended our experiment to 10 English-Chinese bilingual readers.

### Experiment 2: Reading in English-Chinese bilinguals

**Fig. 5.**
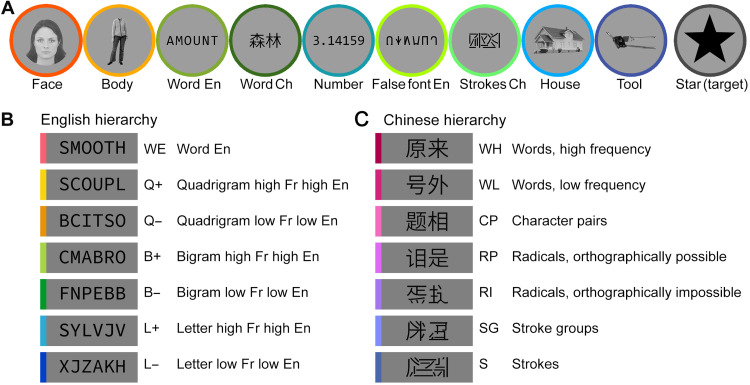
Stimuli and procedure for Experiment 2 with English-Chinese bilingual readers. (**A**) Examples of the nine categories of visual stimuli and the target used in the localizer. Most of the conditions were the same as in the English-French experiment, with word Ch replacing word Fr and strokes Ch replacing Checkerboards. (**B** and **C**) Design of the word-similarity experiment. (B) English stimuli, identical to the corresponding conditions in the English-French experiment (see Fig. 1). (C) Chinese stimuli with hierarchical components increasingly similar to real words (see Materials and Methods for details).

The experimental design was very similar to the English-French experiment (Fig. 5). The localizer used the same design, except that French words and checkerboards were replaced by Chinese words and scrambled strokes derived from these words, respectively. The main fMRI runs consisted of two distinct hierarchies of English and Chinese stimuli with increasing similarity to real words. The English stimuli were taken directly from the English-French main fMRI runs, i.e., the conditions in which the frequencies of the orthographic components were congruent in English and French (L−, L+, B−, B+, Q−, and Q+), as well as the real English words (WE). The Chinese stimuli included strokes covering the entire area where two Chinese characters could appear; strokes organized in two groups similar to two Chinese characters; Chinese radicals arranged to form two pseudo-characters (with radicals arranged in either orthographically impossible or possible positions); real Chinese characters whose pairings formed nonwords; and, last, real Chinese words, two characters long, of low and high frequency (Fig. 5 and see Materials and Methods for details).

#### Localizer for visual categories and overall reading circuit

**Fig. 6.**
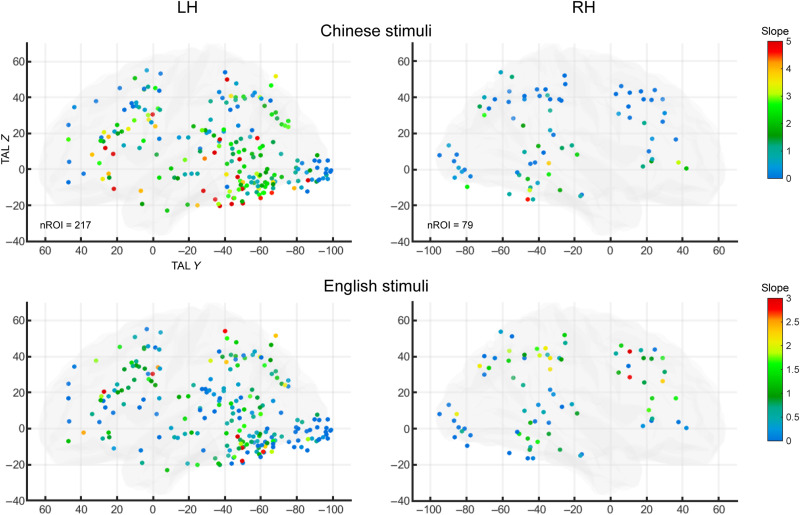
The word-similarity effect in word-specific ROIs of English-Chinese bilingual readers. Same format as Fig. 3A. Word similarity was modeled as the slope of a linear regression over the first five stimulus conditions for each language. Slopes for Chinese and English word-similarity effects are color-coded for each of the 296 ROIs from the contrast of English and Chinese > others in the localizer. Note that the scales for Chinese (top) and English (bottom) stimuli are different.

For consistency, we performed the same bilingual word-specific contrasts as in the English-French study, irrespective of languages (English and Chinese words > faces, bodies, houses, and tools). Similar to the English-French study, the localizer revealed that every individual English-Chinese participant had robust word-specific activation clusters in the VOTC, lateral temporal, and lateral frontal regions (296 ROIs across participants; Fig. 6 and fig. S6). In the VOTC, the mean number of word-specific clusters and voxels was again larger in the left hemisphere (paired *t* tests; 7.9 versus 2.1 clusters, *P* = 0.0079; 496.2 versus 71.7 voxels, *P* = 2.99 × 10^−4^; no right-hemisphere voxel for participants SC05 and SC10). However, the bilingual word-specific contrast overlapped significantly less with voxels from the single language-specific contrasts (whole brain, English: mean = 68.01%, SD = 25.39%; Chinese: mean = 71.70%, SD = 16.12%; VOTC, English: mean = 64.40%, SD = 22.51%; Chinese: mean = 79.90%, SD = 13.04%), compared to the high overlap in the English-French participants (Wilcoxon rank sum test, whole brain: *P* = 4.898 × 10^−4^; VOTC: *P* = 1.482 × 10^−5^). This indicates that the bilingual word-specific contrast in the localizer is likely missing language-specific voxels, and that the two languages are potentially more segregated in the English-Chinese participants.

We also found face-specific clusters in the bilateral VOTC of every participant, although the number of clusters and voxels did not differ significantly between left and right hemispheres (5.2 versus 5.8 clusters, *P* = 0.452; 264.8 versus 352.7 voxels, *P* = 0.252).

#### Spatial gradient of word similarity

Within these bilingual word-specific clusters, we examined the word-similarity slopes and gradients similar to the experiment on English-French bilinguals but now separately for English and Chinese (Fig. 6, comparable to Fig. 3A). We did not perform FDR corrections here because of the much smaller number of conditions being fitted (first 5 conditions per language in Experiment 2 versus all 14 conditions in Experiment 1; see Materials and Methods). Of the 296 ROIs, 74 and 40 showed a significant slope for Chinese and English, respectively, including 23 and 15 ROIs in the VOTC (*P* < 0.05, uncorrected). In the left VOTC, there was a significant increase in the word-similarity effect along the posterior-to-anterior axis for both English and Chinese [word-similarity slopes fitted with TAL *Y* coordinates in individual participants, one-sample *t* test for the resulting regression coefficients against 0; English: *t*(9) = 2.755, *P* = 0.0223; Chinese: *t*(9) = 4.161, *P* = 0.0024]. Note that because the English and Chinese conditions did not have one-to-one correspondence, the word-similarity slopes and their spatial gradients cannot be meaningfully compared between languages.

As in the English-French study, we assessed the hypothesis that discrete cortical patches would be sensitive to different hierarchical levels of stimulus structure. We performed ROI-GLM contrasts in the word-specific ROIs, comparing pairwise conditions. For English stimuli, as in Experiment 1, we found no effect of frequency for letters (L+ > L−), bigrams (B+ > B−), or quadrigrams (Q+ > Q−) but an effect of lexicality effect in lateral temporal and lateral frontal regions. For Chinese stimuli, the pairwise contrasts between the four consecutive noncharacter conditions (SG > S, RI > SG, and RP > RI) each showed significant ROIs distributed along the VOTC, with no evidence that the processing of hierarchical levels are associated with distinct anatomical regions. When contrasting real characters with orthographically possible radicals (CP > RP), significant clusters were mostly located in the lateral temporal and lateral frontal areas, similar to the lexicality effect for English stimuli. Last, other contrasts involving real characters (WL > CP and WH > WL) did not show any significant ROI.

We analyzed the effects of language dominance as in Experiment 1 but did not find any significant correlations in any brain region or subregion (all *P* > 0.084). This may be because the sample size of English-Chinese bilinguals is smaller (*n* = 10) and all of these participants were dominant in Chinese.

#### Cortical patches specialized for Chinese

Different from Experiment 1 with English-French bilinguals, the localizer data for English-Chinese bilinguals are likely missing some language-specific clusters, since the bilingual word-specific contrast overlapped much less with the single language-specific contrasts. To maximize sensitivity and avoid false negatives, we localized them using the data from the main fMRI runs (more data, 15 repetitions versus 5 in the localizer), then replicated, and extended the findings using the independent data from the localizer.

**Fig. 7.**
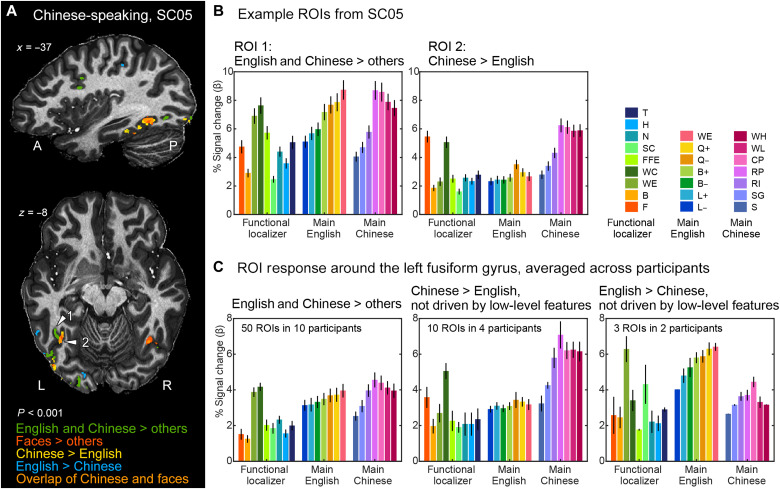
Word-specific and language-specific activation in English-Chinese bilingual readers. (**A**) Example of ventral occipitotemporal activation in a representative participant (SC05; *P* < 0.001 uncorrected; see fig. S6 for the maps of all 10 participants). The word-specific and face-specific clusters were defined with the localizer (English and Chinese words > faces, bodies, houses, and tools; faces > bodies, houses, tools, and English and Chinese words). The Chinese- and English-specific clusters were defined with the main fMRI runs (Chinese words > English words and vice versa). Note the overlapping sensitivity for Chinese words and for faces (orange color) in ROI 2 (white arrowheads) and a symmetrical right-hemispheric ROI. (**B**) Activity profile of two example ROIs from participant SC05, labeled with white arrowheads in (A). ROI 1 shows shared responses to English and French words and corresponding word-similarity effects, while ROI 2 is sensitive to Chinese stimuli and faces. (**C**) Group-averaged activity profiles of bilingual word-specific ROIs around the left fusiform gyrus (left), with preference for Chinese stimuli (middle), or with preference for English stimuli (right). Note the strong response to faces (red bar) in the Chinese-specific ROIs (middle). See figs. S7 and S8 for all individual ROIs.

These analyses revealed robust Chinese-specific clusters in the majority of participants. Contrasting Chinese words (high, low frequency) > English words (*P* < 0.001 uncorrected, cluster size > 4) resulted in 66 bilateral activation clusters, mostly located around the fusiform gyrus (e.g., in OTS and IOS, yellow clusters in Fig. 7 and fig. S6; 31 clusters, 8 of 10 participants) and in the STS (24 clusters, 5 of 10 participants). The fusiform clusters were more numerous in the left than in the right hemisphere (mean: 2.87 versus 1.00 clusters, *P* = 0.0058), which did not differ from the leftward bias observed for the bilingual English-Chinese clusters [χ^2^(1) = 0.758, *P* = 0.3840]. The remaining 11 clusters were from a few participants, including 5 early visual area clusters from three participants (SC05, SC08, and SC10), 5 frontal clusters from two participants (SC04 and SC06), and 1 IPS cluster from SC10. In most clusters, the Chinese specificity was replicated with independent data from the localizer (ROI-GLM contrasts on the localizer time courses): The contrast of Chinese > English words was significant in most clusters across participants (*P* ≤ 0.001 in 31 of 66 clusters and 0.05 > *P* > 0.001 in another 24 of 66 clusters, no FDR correction) and failed to reach significance in only 11 of 66 clusters. As for the Chinese-specific clusters located around the fusiform gyrus (in OTS/IOS/inferior occipital gyrus), the language preference was systematically replicated for 87% (27 of 31) of these clusters in the localizer (all *P* < 0.05, FDR *q* < 0.05).

Focusing on these 31 fusiform Chinese-preferring clusters, we further examined whether the language preference could be explained by low-level retinotopic differences between the stimuli. If this were the case, then the same language difference should be present between the matched low-level control conditions. Therefore, we assessed the interaction (English > Chinese words) − (English false fonts > Chinese strokes) in the localizer. The interaction reached significance in 11 of 31 clusters (10 of which in the left hemisphere of four participants), demonstrating Chinese specificity independent of low-level features (see Fig. 7C for averaged activity profiles across participants and fig. S7 for individual clusters; the latter figure shows that in fusiform/OTS clusters, even when the interaction failed to reach significance, its direction was almost always consistent with a genuine language-specific effect).

The fusiform clusters showed further signatures of selective tuning to the Chinese script in the word-similarity slopes. The Chinese-specific clusters showed a significant word-similarity slope only across Chinese conditions, but not across English conditions (*P* < 0.05 in 12 of 31 ROIs for Chinese and 0 of 31 ROIs for English, no FDR correction to avoid false negatives). Visual inspection confirmed that most fusiform ROIs had a distinctive activity profile characterized by the following: (i) an almost flat activity profile across the seven conditions with variable similarity to English words; (ii) an increasing activity across the first four conditions with variable similarity to Chinese words; and (iii) a high activity for the last four conditions, i.e., for all orthographically possible combinations of Chinese radicals, with a trend for a decrease in activity for real characters and words. This finding indicates that these regions, much like the classical VWFA, are prelexical in nature and can be strongly activated by pseudo-words with the right kind of subunits.

The localizer also led to an unexpected finding of overlap between face-specific and Chinese-specific activations (Fig. 7A). While Chinese-specific VOTC clusters showed a much higher activity for Chinese words than for almost all other categories in the localizer, including English words, there was one exception: They were strongly activated by faces (Fig. 7 and fig. S7). ROIs (25 of 31) showed significant face specificity in the ROI-GLM (faces > bodies, houses, and tools; 14 ROIs with *P* ≤ 0.001 and 11 ROIs with 0.001 < *P* < 0.05). This specificity for faces remained true in the subset of clusters whose Chinese specificity was demonstrably not driven by low-level features (Fig. 7C, middle). Furthermore, Chinese-specific clusters often overlapped or were very close to face-specific clusters (fig. S6, yellow arrowheads).

#### Very few cortical patches specialized for English

In the opposite direction, for the contrast of English > Chinese words, one participant did not have a single English-specific cluster (SC06) and another participant had an abnormally large number of them (SC03: >200 clusters at *P* < 0.001 and 46 clusters even at a more stringent threshold of *P* < 0.0001, mainly in frontal, early visual area, and middle occipital gyrus). Aside from these two outliers, the majority of the clusters for this English-specific contrast for the remaining eight participants was consistently found in early visual areas (23 of 62 clusters) and was due to visual rather than linguistic preference, as 22 of 23 did not meet the criterion of showing a critical interaction relative to lower-level stimuli (Chinese > English words) − (Chinese strokes > English false fonts). Other clusters were found in the OTS/IOS region (11 of 62 clusters in five participants; fig. S8), and 5 of 11 of them passed the critical interaction for English specificity not driven by low-level differences (fig. S8, A and C, three clusters in the left hemisphere). These five clusters therefore qualified for English specificity, but three of them showed a significant word-similarity slope not only for English but also for Chinese (*P* < 0.05, no FDR correction). These five clusters came from only two participants, who had neither the highest English language dominance score nor the highest number of English words read per minute. The remaining 28 of 62 English-preferring clusters came from only three participants and were mainly located in the IPS and lateral frontal areas. In summary, as compared to Chinese-specific activations, English-specific clusters were few, even fewer in the VOTC, and their language specificity was weak.

### Data-driven comparison of English-French and English-Chinese bilinguals

Looking for evidence of language specialization in the VOTC requires pushing the spatial resolution as much as possible, which is why we used 7-T imaging at 1.2 mm isotropic. Nevertheless, each of our voxels contained ~150,000 to 200,000 neurons with different functional properties; furthermore, to achieve adequate SNR, many of the above analyses averaged the signal over multiple voxels that we implicitly assumed to be homogeneous within each cluster. In a final analysis, we tried to sidestep this limitation using “hypothesis-free decomposition” (*35*), a nonparametric independent component analysis (ICA) that can infer the multiple canonical response profiles of distinct neural populations that may overlap within the same voxel. This method was previously shown to identify distinct neural responses to music and to speech in 3-T fMRI (*35*), which was later replicated and extended by direct intracranial analysis (*36*).

We used this approach to decompose the response profiles from the main fMRI runs, within all the bilingual word-specific VOTC voxels defined by the localizer data (Experiment 1: English and French words > others; Experiment 2: English and Chinese words > others; *P* < 0.001 uncorrected, cluster size > 4). For each group of bilinguals, we decomposed the 14 conditions into three canonical response profiles, i.e., the minimum number required to model baseline activity, the word-similarity effect, and putative language differences. Activity in each voxel was expressed as a weighted sum of these three profiles, each multiplied by a voxel-specific weight.

**Fig. 8.**
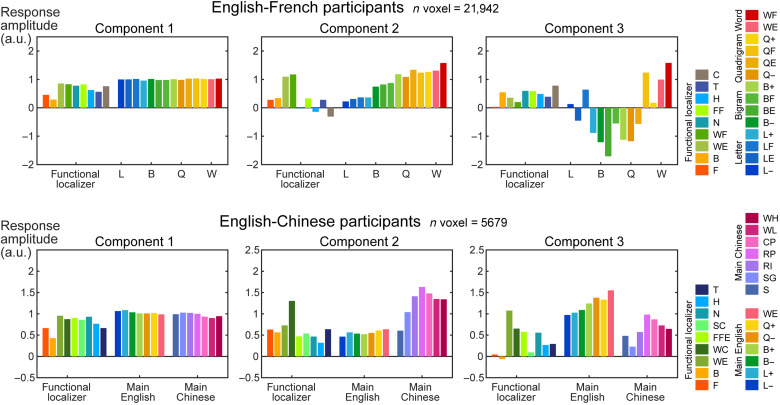
Data-driven decomposition of activity profiles in VOTC. A variant of ICA was applied to the activity profiles of the main fMRI runs (14 rightmost bars in each graph) within each word-specific voxel identified in the localizer. The resulting weights were also applied to the localizer data to derive their activity profile (leftmost bars). For English-French participants, activity was decomposed into a weighted sum of constant, linear, and nonlinear shapes of the word-similarity effect, with no clear separation between English and French stimuli. For English-Chinese participants, by contrast, components 2 and 3 clearly showed distinct preferences for either Chinese or English stimuli.

Figure 8 shows the data-driven component profiles that were identified by this procedure. Although the components were identified solely from the 14 rightmost conditions (main fMRI runs only), for completeness, the figure also shows the corresponding activity profiles from the localizer run, which were obtained by applying to each voxel the component weights from the main fMRI runs. In both language groups, we observed a flat component showing similar activity amplitude across all conditions, similar to the activity in EVC (compare Figs. 8 and 3B). The other components confirmed that English-French and English-Chinese bilinguals differed sharply. For English-French participants, the second component showed an overall word-similarity effect that was almost perfectly continuous and monotonic, with no differences between languages. The third component was noisy and showed an overall quadratic organization, presumably capturing between-voxel variations in the shape of the word-similarity effect. Although it did show hints of language differences (higher amplitude for LF, BF, QF, and WF than for LE, BE, QE, and WE, respectively), this pattern was not consistent with the localizer profile, where WE was higher than WF, i.e., in the opposite direction.

The results were notably different for English-Chinese participants (Fig. 8, bottom row). The word-similarity effect was decomposed into two distinct components, one for English (component 3) and one for Chinese (component 2). Both showed a higher amplitude for stimuli in the preferred language, regardless of whether they were words or pseudo-words. Furthermore, this language difference was replicated in the localizer profiles. These language differences were not driven solely by the Chinese- and English-specific voxels. First, only a small fraction (404 and 257 of 5679 voxels, 11% in total) of the word-specific voxels analyzed here (as defined by the localizer data) overlapped with the Chinese- or English-specific voxels (as defined by the main fMRI runs). Second, removing these language-specific voxels did not qualitatively change the shape of the component profiles. In summary, these analyses demonstrated the added value of a purely data-driven ICA analysis: This analysis was able to identify subvoxel functional properties and confirmed the presence of a language specialization in English-Chinese, but not in English-French, participants.

## DISCUSSION

In the current 7-T fMRI study, we examined the organization of reading-related cortical patches in English-French and English-Chinese bilinguals. Because we operated at a high resolution and SNR, our analyses were entirely focused on individual participants. Despite interindividual variability, three general conclusions can be drawn: (i) The VWFA actually consists of multiple cortical patches with high selectivity for written words; (ii) identical patches respond to English and French stimuli in bilingual English-French readers, but partially distinct patches respond to Chinese and English scripts in bilingual English-Chinese readers; (iii) the patches are highly sensitive to language statistics. We discuss these points in turn.

### Multiple specialized cortical patches for reading

By analyzing unsmoothed fMRI data at 1.2-mm isotropic resolution, we found that what previously appeared, at a lower resolution, as a single extended VWFA actually consists of a multiplicity of small and highly specialized word-specific patches distributed in the VOTC along the OTS/IOS. These reading-related patches bear similarity to the face patches found both in the current study and in previous literature, although showing perhaps less stereotypical distribution across participants (*29*, *37*). For both English-French and English-Chinese participants, word patches are present in both hemispheres but with a left-hemisphere bias in the number of clusters and voxels.

We largely prevented the usual signal drop in the anterior inferior temporal lobe (between TAL *Y* = −50 and −22) and found that this region also contains cortical patches with high word selectivity. These clusters may correspond to the supramodal “basal temporal language area” in the intracranial recording literature (*38*–*40*). In a recent intracranial study, the word-selective sites anterior to TAL *Y* = −40 were more responsive to words than to false fonts and infrequent letters under a passive viewing task (*16*), consistent with our results (fig. S4).

Given the numerous word patches found along the OTS/IOS and the difficulty to arrange them into larger groups reproducible across participants, the word patches may be better studied individually. By analyzing them one by one, we identified several local cortical properties. First, word patches can be exquisitely specialized, showing much lower activity for nonword stimuli with similar low-level features, such as numbers, false fonts, and character strokes, and also for other object categories with less similar visual features (bodies, houses, and tools). In this respect, 7-T fMRI converges with intracranial recordings in suggesting that the VOTC is a patchwork of small, highly specific cortical sectors each responsive to a small category of stimuli, including learned ones such as letter or number strings (*27*, *31*, *41*). An analogy to face patches suggests that word patches may contain a vast majority of neurons highly specialized for letters and written words (*42*)—a prediction that may become testable as high-density single-cell recordings (e.g., Neuropixels) become available in humans. Supporting this prediction, simulations of neural networks designed for object and face recognition but recycled for written word recognition show that a small proportion of units becomes dedicated entirely to reading (*12*).

However, an unexpected finding was that many of the Chinese-specific patches also showed high face specificity (faces > bodies, houses, and tools) [for related findings, see (*43*, *44*). Chinese characters may share with faces a need for holistic (or configural) processing (*45*). While alphabetic writing also calls for encoding the relative position of letters (*46*, *47*), information may be more configural in Chinese characters [for a review, see (*48*)], which exhibit symmetries, horizontal and vertical alignments, and other regularities in two dimensions. In addition, the shared overall round shapes of Chinese characters and faces may be preferred by a ventral visual network for “stubby” shapes, as reported in monkeys (*49*).

The cortical overlap between Chinese characters and faces is reminiscent of the frequent interactions that have been observed between reading and face recognition during the acquisition of literacy. When comparing literate and illiterate participants, the activity evoked by faces tended to decrease with literacy in the left-hemisphere VWFA location and showed a strong increase in the right hemisphere (*4*). This previous finding suggested a competition between the newly acquired words and the already existing face specificity, consistent with the cultural recycling hypothesis (*50*). The word-face competition hypothesis was revisited in later developmental studies of children learning to read (*3*, *51*): During reading acquisition, the VWFA invades largely unspecialized cortical patches in the left OTS, and their growth blocks the slow development of face patches, forcing them to shift toward the right hemisphere (*51*, *52*), perhaps because both call upon the same cytoarchitectonic region (*53*). The current study confirms that there may indeed be shared mechanisms between face and word processing but unexpectedly indicates that cortical competition (in alphabetic readers) may switch to cortical overlap (in Chinese readers). Whether this overlap is reflected in a performance enhancement (potential skill transfer) should be clarified in future studies.

### Script-specific patches

The main goal of our study was to ask whether the VWFA splits in bilingual readers. We found that the answer depends on whether the languages share the same script. For English versus Chinese, we found a partial split: Several VOTC clusters were highly specific for Chinese only, showing the word-similarity effect only for Chinese, while very few showed the opposite preference for English over Chinese. Even within voxels selected for their reading-related activity regardless of language, a data-driven analysis indicated the presence of subvoxel preferences for one or the other language (Fig. 8). However, there was no such separation when English and French stimuli were contrasted in English-French bilinguals. One may ask whether the existence of Chinese-specific activations in English-Chinese bilinguals resulted from Chinese being the dominant or first language and not from its specific orthographic features. If this were the case, however, we should find French-specific activations in French-dominant participants and English-specific activations in English-dominant participants, which was not the case. In the same vein, we ensured that the existence of only Chinese-specific activation was not due to the differences in the age of second-language acquisition between Chinese-, English-, and French-dominant participants. As shown in Materials and Methods, the age of acquisition (AoA) did not differ between these subgroups. The absence of a language split in the English-French group is also unlikely to be due to lack of power, as we had 21 participants in the English-French group, twice the size of the English-Chinese group. The same word-similarity slope for both English and French conditions could be observed for individual participants, ROIs, and even single 1.2-mm voxels, with no consistent language bias. In reading-related clusters around the fusiform area, we even detected a subtle dominance effect across participants, where the activity balance between English and French words was in favor of the less dominant language (fig. S5), which we interpret as greater top-down attention or cognitive effort. In the subvoxel profiles, the number of voxels analyzed for the English-French group was also almost four times as numerous as for the English-Chinese group, yet no language split was detected. This lack of English-French differences is in agreement with the bilingual interactive activation model of bilingual language processing (*18*, *54*), which posits that word identification is fully integrated across languages, including shared orthographic processing up to lexical access. Only then are word identities mapped onto language membership, which may then control relative activity in the representations of the two languages. This late control may account for the subtle language dominance effect that we observed, showing higher activation for real words in the nondominant language.

The discovery of Chinese-specific voxels here was made possible by the small voxel size (1.2 mm isotropic) and high SNR of 7-T fMRI. Using coarser resolution, previous studies mostly failed to find differences between the cortical circuits for reading, both between languages using the same alphabet and between Chinese/Japanese and alphabetical languages (*19*). This is notably true for VOTC activations both in monolingual and bilingual individuals (*19*, *20*, *55*). Still, time-resolved methods may be more sensitive to differences between scripts. Using electroencephalography in monolingual participants, Yum *et al.* (*56*) found a left-lateralized N170 component, originating from VOTC, which differed between English participants reading English words and Chinese participants reading Chinese characters. An electroencephalography/magnetoencephalography version of the present study could clarify the time course of the divergence between scripts. Using fMRI, several authors found that, at the group level, Chinese/Japanese character recognition induces slightly more right-lateralized VOTC activations than alphabetical reading (*6*, *57*–*59*). We did not find such a rightward bias, possibly because our participants were native Chinese speakers, while the rightward bias may be specific to logographic scripts acquired as a second language [for a review, see (*60*)].

More generally, in the literature on bilingual reading, a tension between assimilation and accommodation has been proposed for the learning of the first (L1) and second (L2) languages (*61*). When L2 is similar to L1, the brain would use the same L1 network for reading L2 (assimilation). This has been shown in fMRI studies of English and Korean bilinguals where both languages are alphabetical [e.g., (*62*)]. When L2 is widely different from L1, the brain would use new areas for reading L2 (accommodation). This has been suggested to occur in English L1 participants learning Chinese as L2, including in the right fusiform region (*61*, *63*), middle occipital gyri (*62*), and middle frontal gyrus (*63*). Our results are partially in line with this theory that there is still much assimilation in English-Chinese readers, as many voxels show similar responses and word-similarity effects for both languages.

We see two reasons for the partially distinct cortical specialization in English-Chinese readers. The first hypothesis is that the visual features of Chinese characters differ from those of the Roman alphabet used for English and French. While all writing systems share similar graphic principles and appeal to similar line intersections (*23*, *64*), they differ in the nature, number, and spatial arrangement of the shapes used, which may require a dedicated set of neural circuits. In support of this idea, we recently showed that, when recycling a convolutional neural network to recognize 1000 written French words, the network dedicates a few dozen units to letter shapes (*12*). In new simulations, we trained the same network to recognize either 500 English and 500 Chinese words or 500 English and 500 French words, thus providing an elementary simulation of bilingual reading (fig. S9). The English-French network developed 54 word-selective units (±5 across replications), none of which were selective to a specific language. However, the English-Chinese network developed 114 (±5) word-selective units, of which 26 (±5) were selective to Chinese characters and 14 (±4) to the alphabet. Thus, an artificial network driven solely by the needs of invariant visual recognition finds it advantageous to dedicate some unique processing resources to the shapes of Chinese and alphabetic writing. It seems likely that the same constraints on invariant visual recognition would apply to the cortex.

However, visual shape alone is unlikely to fully explain ventral visual cortex specialization because Arabic numerals share visual features with letters and yet is processed in a specialized visual number area lateral to the OTS/IFS (*65*, *66*). Moreover, there is a strong overlap of the VOTC regions activated during visual reading and during reading through tactile or auditory channels in blind participants in previous studies at 3 T (*67*, *68*). Thus, an alternative possibility is that the localization of the VWFA is strongly influenced by the prior connectivity of the cortex to other areas (*3*, *9*, *10*, *69*). Since Chinese is a less phonologically transparent language as compared to English and French, it may place greater demands on the direct lexical route, thus relying on a slightly different set of connections and therefore, putatively, a different preferred site of origin in ventral visual cortex (*70*).

A limitation of the present study is that we contrasted maximally different logographic and alphabetic writing systems (English versus Chinese) and minimally different systems using the same alphabet (English versus French). This leaves open a number of questions: (i) What should we predict for the intermediate case of languages using different alphabetic scripts, such as English versus Russian or Hindi? If the English-Chinese dissociation resulted entirely from the gross geometrical differences between the two scripts, then we should expect no difference between two visually comparable alphabets. However, if the dissociation is due to the impossibility of computing pooled statistics over two disjoint sets of letters, then a dissociation could be observed. (ii) Would the VOTC also split its resources in bilingual readers mastering two languages with the same alphabet but very different letter statistics, such as French and Polish? (iii) Does the direction of reading matter, as could be studied in Arabic-French or Hebrew-English bilinguals? Answering these questions, possibly using the present study as a template, would shed light on the causal factors underlying the cortical specialization for reading, but we should keep in mind the difficulty of recruiting a large group of fluent bilingual volunteers for a 7-T fMRI study.

### Sensitivity to the statistics of written language

Last, the current study underlines the replicability of the word-similarity effect (*15*), not only in English-French alphabets but also in Chinese [as recently reported in (*71*)]. When written stimuli respect increasingly well the statistics of words in the known language, they elicit increasingly greater activity in the VWFA (*72*). Many word-specific clusters in English-Chinese participants even shared similar word-similarity profiles for English and Chinese. The present findings suggest that this property is not a general feature of the entire VOTC, as the heavily smoothed group-level images of Vinckier *et al.* (*15*) may have suggested, but is a very local feature unique to word-specific patches, which is absent e.g., in face-responsive patches that are not immediately adjacent to the word-responsive patches.

Two complementary mechanisms may give rise to this effect. First, in a bottom-up manner, during reading acquisition, the visual cortex may incorporate the statistics of the shapes (e.g., letters and letter groups) that make up words. Imaging data (*5*, *73*) and simulations (*12*) show how visual neural networks become progressively tuned to the specific shapes of letters and their combinations that are useful for recognizing words in a specific script. As a result, already during the initial bottom-up wave of VOTC activation, the cortex would be increasingly responsive to letter strings that increasingly mimic the statistics of real words. The second, not-necessarily exclusive possibility is a top-down mechanism: The visual inputs that are more similar to real words may be processed at a higher level, for example, evoking multiple candidates within the lexicon, and thereby elicit more top-down information from higher brain regions back to visual cortex (*74*).

Because of its poor dynamic resolution, fMRI is largely unable to separate these two possibilities. However, intracranial recordings suggest that both mechanisms may contribute, at different times following stimulus onset (*16*). Broadband gamma activity in the left VOTC shows a larger initial peak (~250 ms) when stimuli are more similar to real words (*16*), suggesting a bottom-up effect. However, this early effect primarily separates stimuli based on their letter frequency (frequent versus infrequent letters), suggesting that the bottom-up wave may primarily compile letter-based statistics. The effect of larger orthographic units, i.e., bigrams and quadrigrams, only appears later and in a top-down manner, seemingly triggered by a lexical effect that first emerges in the anterior fusiform (*16*, *38*).

Under the latter interpretation, while word-specific VOTC would be genuinely tuned to letters [contra (*75*)], most of the continuous, monotonic word-similarity effect seen here with fMRI would be due to top-down signals proportional to the strength of lexical activation. Such an interpretation is compatible with (i) simulations of purely bottom-up neural networks for reading, which only show a difference between frequent and infrequent letters and not the full Vinckier *et al.* (*15*) word-similarity effect (*12*); (ii) evidence for a reversal of lexicality effects in VOTC when the task changes from passive viewing to active sentence reading, highlighting the influence of top-down processing on these activity profiles (*16*, *76*); (iii) evidence for top-down activation of VOTC during spoken language processing (*4*, *38*); and (iv) the present evidence for greater VOTC activity for the nondominant language in English-French bilinguals, which we interpret as a top-down effect due to greater attention and cognitive effort.

In conclusion, the present results illustrate the power of cortical plasticity and education in shaping the fine details of the human visual cortex. Not only do localized patches of cortex, at a millimeter scale, become highly tuned to the statistics of words and word-like stimuli in the learned script, but they may also even become tuned to a single script in bilingual readers, at least when those scripts differ sufficiently at the visual level.

## MATERIALS AND METHODS

### Participants

Twenty-one English-French bilinguals and 10 English-Chinese bilinguals were recruited from the Paris region and participated in this study. The 21 English-French participants consisted of three subgroups (seven participants each): a balanced early bilingual subgroup (age range = 18 to 35, mean age = 22.7, four females), where participants grew up in bilingual environments and were native speakers/readers of both English and French, having acquired both languages before the age of 10; an English-dominant subgroup (age range = 21 to 32, mean age = 25.4, two females); and a French-dominant subgroup (age range = 20 to 26, mean age = 22.1, four females). In the latter two subgroups, participants acquired one of the languages as their native language and later learned the other language at school, so that they became fluent readers (see below).

Given our testing site (NeuroSpin, near Paris) and the fact that these experiments took place during the coronavirus disease epidemic, we could not compose similar subgroups for English-Chinese bilinguals (participants who learn Chinese as a second language at school and yet become fluent readers are quite rare). Instead, the 10 English-Chinese participants (age range = 20 to 31, mean age = 25.7, seven female) were all native speakers/readers of simplified Chinese who later became fluent in English.

All participants were assigned to groups according to their self-reports and the Language Experience and Proficiency Questionnaire (*77*) that they filled in during recruitment. The self-reported age at which they started acquiring each language (AoA) is listed in table S1. Consistent with our recruitment criteria, the AoA differed between the first and second languages within each subgroup of late bilinguals, including the English-dominant and French-dominant participants from Experiment 1 and the Chinese-dominant participants from Experiment 2 (*n* = 7, 7, and 10, respectively; Wilcoxon rank sum test within each group, all *P* < 0.0027). We also compared the AoA of the second language between these three subgroups of late bilinguals. There were no significant pairwise differences between groups in either the spoken (Wilcoxon rank sum test, all *P* > 0.162) or written (Wilcoxon rank sum test, all *P* > 0.592) modality. Participants also completed an online language test for English and French (https://dialangweb.lancaster.ac.uk/getals; tests on listening and structure, the English-Chinese participants only completed the English test). All achieved at least B2 level. A few participants were also familiar with a third/fourth language, although the proficiency was always at a much lower level compared to the three languages tested in the current study.

All participants were right-handed, had normal or corrected-to-normal vision, and had no history of neurological/psychiatric disorders or reading/learning difficulties. Participants provided written informed consent for the fMRI study and received monetary compensation. The study was approved by the local ethics committee in the NeuroSpin Center (CPP 100032 and 100055), and the study was conducted in accordance with the Declaration of Helsinki.

### Stimuli

#### Functional localizers

The stimuli for the English-French functional localizer (hereafter referred to as “localizer”; Fig. 1A) were gray-scale images from eight different object categories (20 exemplars per category). They were adapted from stimulus databases and previous experiments [see stimuli and details in the Open Science Framwork (OSF) data repository], including faces (neutral, 10 males), bodies without heads (neutral standing still, 10 males), English words (*78*), French words (www.lexique.org/) (*79*), numbers (famous mathematical constants, six to seven digits, including the decimal point and + and − signs), false fonts (six letters; no letter was repeated on the same position) (*15*), houses, and tools (half in a position graspable by the left hand). Both English and French words were six-letter strings, with word frequencies (computed from movie subtitles) ranging from 7 to 761 per million. Words and numbers were rendered in the Consolas font. All stimuli had a width or height of <227 pixels (the longer axis of <241 pixels) and were embedded within a gray circle (RGB: 157, 157, 157; diameter = 310 pixels, 3.2°) presented in the center of a black screen. Stimuli were controlled across conditions for mean luminance and mean root mean square contrast within the circle. Under an additional checkerboard condition, two alternating checkerboards of opposite pixel intensities were presented at the same temporal rate as the other stimulus categories, filling in the entire gray circle. A black star served as the target, and participants had to press a button on a cylinder button box with their right thumb as soon as they detected it.

Most of the conditions in the English-Chinese localizer were identical to those in the English-French localizer. Only the French words were replaced by Chinese two-character real words (rendered in the Source Han Mono font, which is very similar to Consolas, word frequency matched to the English words) (*80*), and the checkerboards (not used in the analysis of the English-French experiment) were replaced by the single strokes decomposed from the Chinese real words.

#### Main fMRI runs

##### English-French stimuli

To build the English-French materials, we first computed the (log) frequencies of letters, bigrams, and quadrigrams in English and French, using lexical frequency information from the SUBTLEX field from the British Lexicon Project database (http://crr.ugent.be/programs-data/lexicon-projects) (*78*) and the FREQFILMS field from the French Lexique database (version 3.82; www.lexique.org/) (*79*).

Second, we generated all possible six-letter strings using the 26 letters of the alphabet (ignoring French accented letters). We then excluded all strings that did not contain a single quadrigram present in either French or English, reducing the corpus by a factor of ~10. To avoid spurious lexical effects, we also removed strings in which five-letter words were embedded. For each remaining six-letter string, we obtained the average frequencies of its letters, bigrams, and quadrigrams, in English and French.

From this set of six-letter strings, we created 14 categories of stimuli, each comprising 180 items (Fig. 1B). Four categories, devoted to the study of letter frequency, resulted from the crossing of French statistics (strings with high- versus low-frequency letters) × English statistics (strings with high- versus low-frequency letters). Four similarly crossed categories were devoted to the study of bigram frequency, and four others to the study of quadrigram frequency. All stimuli, so far, were nonwords. The last two categories, devoted to the study of lexicality, consisted of real French and English words.

For the 12 sublexical-component conditions, we used only nonwords and selected sets of stimuli so that frequency should not be correlated across types of sublexical components (Fig. 1C). For example, we wanted to ensure that effects of quadrigram frequency could not result from effects of bigram frequency, although the frequency of quadrigrams and bigrams tend to be correlated in random letter strings. The selection of stimuli was optimized on the basis of the average frequencies of their component letters, bigrams, and quadrigrams (Fig. 1C).

For the two lexical conditions, we selected French words that were not English words and vice versa. These words were matched in all respects (frequency of letters, bigrams, and quadrigrams) with stimuli from the quadrigram categories: English words with items with a high frequency of quadrigrams in English and a low frequency in French and vice versa for French words. This amounted to comparing real French words to French-looking pseudo-words and the same for English.

The code for generating the stimuli can be found at the stimuli folder of the repository: https://osf.io/96syx/?view_only=88b55034027042fbb4f118f398fc5706. All stimuli were rendered during the experiment using the monospaced Consolas font at 66 points.

#### English-Chinese stimuli

##### English stimuli

The seven English stimulus conditions were taken from the English-French experiment, i.e., six word component conditions with low or high frequencies in both languages (L−, L+, B−, B+, Q−, and Q+) and English real words.

##### Chinese stimuli

In the Chinese writing system, words are formed by one or more characters (in a specific order; changing the order often changes the meaning of the word). The characters themselves are composed of one or several radicals (graphical components) in various orthographic compositions (e.g., left-right and up-down). Some radicals are themselves compounds of radicals that can be further decomposed. Last, all radicals can themselves be decomposed into several elementary writing strokes. Given this organization, in analogy to the English and French stimuli, the first author (M.Z.), a native speaker of Chinese, generated a hierarchy of seven Chinese stimulus conditions that all spanned the space of two Chinese characters but were increasingly similar to real Chinese words. Each condition contained 180 stimuli. Note, however, that the seven Chinese conditions were not directly parallel to the seven English conditions.

The top three conditions in this hierarchy (WH, WL, and CP; see Fig. 5) all contained real Chinese characters with matched frequencies. We constructed 180 high-frequency two-character real Chinese words (condition WH; log_10_ frequency = 1.5 to 2, mean = 1.744) from 196 unique characters (all high frequency, log_10_ frequency ranging from 2.5 to 3.5). The low-frequency real words (condition WL; log_10_ frequency = −0.5 to 0, mean = −0.217) were then composed of 158 of the 196 abovementioned unique characters. Last, the character pair condition (CP) was generated from the 185 of the 196 unique characters in condition WH, excluding combinations that (i) were real words, (ii) would become a real word if the two characters were swapped, (iii) had a pronunciation similar to a real word, and (iv) were often contiguous characters in everyday texts (e.g., two connecting characters from two adjacent words).

Two other conditions contain Chinese radicals in orthographically possible (condition RP) and impossible (condition RI) positions, respectively. Condition RP was generated by decomposing all 360 real characters from condition CP into radicals and reassembling them in orthographically possible positions, with a slight change in visual form where appropriate (in Chinese calligraphy and typography, the form of a given radical can change subtly when used in different sizes and positions; e.g., the characters 石磊蠹 share the same radical 石 in different positions). For Chinese readers, the resulting RP stimuli were perceived as extremely low-frequency real Chinese characters that did not remind them of common real words. We verified that the RP stimuli were absent from the Chinese GB2312, GBK, or GB18030 encoding standards (~21,887 ideographs), the CJK glyph database of the Source Han mono font (48,966 ideographs), and the character decomposition series in zh.wiktionary.org but could exist in Unicode’s CJK Unified Ideographs Extensions (92,856 ideographs), which includes non-Chinese (Japanese or Korean) characters.

The RI condition contained exactly the same number and types of radicals as the condition RP, although shifted into orthographically impossible positions. They were created with the following guidelines. Whenever possible, radicals were placed in positions where they cannot occur in the Chinese script or only occur with very low probability, i.e., very low character frequency. For “compound radicals” that can still be further decomposed into subcomponents, e.g., two radicals, the subcomponents themselves were swapped (but such a reconstructed compound radical was occasionally kept in its original position). Last, the characters that we chose contained some high-frequency radicals that can appear in any possible position (e.g., the radical 口, which appears in 吅吕品㗊哀回器…, covering all orthographically possible positions; several other radicals with similar properties include 日又由火木且求). In most of these cases, the positions for these radicals were left blank; in a few cases, we chose a specific visual form of the radical (in terms of calligraphy and typography) that could not appear in the chosen position.

Last, the lowest-level conditions (S and SG) comprised only elementary strokes, directly decomposed from the high-frequency real-word characters (WH). For the first condition (S: strokes), all 360 characters of condition WH were decomposed into Chinese GB13000.1-standard strokes in their natural forms, following the rules of Chinese handwriting (recovering the intersections and corners formed by overlapping strokes, no artificial stroke breaks, and no stroke rotation). The strokes were rearranged so that they spanned the same overall two-character space, without intersecting, and without forming two blocks. The second condition of stroke groups (SG) contained the same strokes, except that the strokes formed two-character–like blocks but without forming any real Chinese radicals or characters (at least in their exact visual forms in the Source Han mono font). The number of crossings, connections, and overlapping corners was kept very similar to the high-frequency real-word condition (WH).

##### Rendering

The English and Chinese stimuli were both rendered in the monospaced font Source Han Mono (https://ccjktype.fonts.adobe.com/2019/05/source-han-mono-v1001.html), whose strokes are visually similar to the alphabetic font Consolas. The English fonts were rendered at 56 points and regular weight (corresponding to the size of 66 points in Consolas), and the Chinese fonts were rendered at 71.27 points and normal weight, so that the stroke widths and spatial areas covered by the two characters were roughly matched to the six-letter English strings.

The Chinese stimuli were decomposed and recombined into vector shapes in Adobe Illustrator CS 6.0. All stimuli were converted to images to ensure that the font rendering was exactly the same for all participants. We controlled for mean pixel values within the English and Chinese conditions. For the first three English conditions, we slightly changed the black pixel values (RGB 0, 0, 0 to 2, 2, 2) for condition L− and made some of the anti-aliased edge pixels on the letters slightly darker for conditions L+ and B−. This resulted in matched mean pixel values across all English conditions, with no noticeable change in the visual percept. For the first four Chinese conditions, we made the strokes 1 pixel thinner for the same reason.

### Data acquisition

Brain images were acquired using a 7-T Magnetom scanner (Siemens, Erlangen, Germany) with a 1Tx/32Rx head coil (Nova Medical, Wilmington, USA) at the NeuroSpin Center of the French Alternative Energies and Atomic Energy Commission. Dielectric pads were used for 30 of 31 participants (not used for SE01 due to insufficient space inside the head coil). To minimize head movements, a tape (padded with tissue paper for comfort) was attached to both the forehead of each participant and the head coil to provide tactile feedback to the participants whenever they attempted to move their head. To minimize light reflections inside the head coil, a piece of black paper was inserted to cover the inner surface of the transmitter coil element. Stimuli were presented on a BOLDscreen 32 LCD screen (Cambridge Research Systems, Rochester, UK; 69.84 × 39.29 cm, resolution = 1920 × 1080 pixels, refresh rate = 120 Hz, viewing distance = ~200 cm), at the head-end of the scanner bore. Participants viewed the screen through a mirror attached to the head coil. The entire scanning session lasted approximately 90 min.

Within each experiment, one localizer run (9 min and 12 s, 276 volumes) and three main fMRI runs (13 min and 18 s per run, 399 volumes) were acquired (because of scanner technical issues, only two main fMRI runs were acquired for participant SB01). Functional data were acquired with a two-dimensional (2D) gradient-echo echo-planar imaging (EPI) sequence [repetition time (TR) = 2000 ms, echo time (TE) = 21 ms, voxel size = 1.2 mm isotropic, multiband acceleration factor = 2; encoding direction: anterior to posterior, iPAT = 3, flip angle = 75, partial Fourier = 6/8, bandwidth = 1488 Hz per pixel, echo spacing = 0.78 ms, number of slices = 70, no gap, reference scan mode: GRE, MB LeakBlock kernel: off, fat suppression enabled]. To correct for EPI distortion, a five-volume functional run with exactly the same parameters except for opposite phase encoding direction (posterior to anterior) was acquired immediately before each task run. Participants were instructed not to move between these two runs. Manual interactive shimming of the B0 field was performed for all participants. The system voltage was set at 250 V for all sessions, and the fat suppression was decreased per run to ensure that the specific absorption rate did not surpass 62% for all functional runs. To minimize artifacts and increase the SNR around the VOTC, for 28 of 31 participants, the functional data acquisition slab was placed to exclude the eyes and the ear canal signal dropout region, so that the VOTC, especially the anterior OTS above the ear canal, was covered as much as possible (see fig. S1). The ear canal signal dropout only affected the anterior VOTC data of the first three participants (SB01, SB02, and SE01) but did not affect the more posterior location of the classical VWFA.

High-resolution MP2RAGE anatomical images were obtained after two or three functional runs (resolution = 0.65 mm isotropic, TR = 5000 ms, TE = 2.51 ms, TI1/TI2 = 900/2750 ms, flip angles = 5/3, iPAT = 2, bandwidth = 250 Hz/Px, echo spacing = 7 ms).

### Experimental design

#### Functional tasks

Both the localizer and the main fMRI runs used a miniblock design in which multiple stimuli were presented rapidly in each block. A green fixation dot (RGB: 112, 219, 96, dot diameter = 8 pixels) was always present in the center of the screen. Participants had to detect a rare target, which, in some blocks, randomly replaced a stimulus after the fifth within a block.

For the localizer, each block contained 20 stimuli and lasted for 6 s, followed by a jittered fixation period of 4, 6, or 8 s (mean = 6 s). Within each block, the stimuli were presented for 100 ms, followed by a fixation period of 200 ms. Each of the nine experimental conditions was repeated five times, two of which contained catch trial targets, except the checkerboard condition that did not contain catch trials. The target of the catch trials was a star. Additional fixation periods of 6 s were added at the beginning and the end of each run.

For both the English-French and English-Chinese main fMRI runs, each block contained 12 stimuli and lasted for 4.2 s, followed by a jittered fixation period of 3.8, 5.8, or 7.8 s (mean = 5.8 s). Within each block, the stimuli were presented for 150 ms, followed by a fixation period of 200 ms. Each of the 14 experimental conditions was repeated 15 times, randomized, and counterbalanced within three runs (five repetitions per run, two of which contained a catch trial target). Across languages, the target was always a string of six hashtags (######). The stimuli within each condition were presented only once, and the presentation order within each block was fixed to avoid the same letter appearing in the same position in consecutive stimuli for the English-French experiment. Each run contained 70 blocks of stimuli, and an additional 7 blocks of fixation-only blocks (jittered between 8, 10, and 12 s, mean = 9.71 s). Fixation periods of 16 and 14 s were added at the beginning and the end of each run. The background color of the screen was gray (RGB: 128, 128, 128).

#### One-minute word reading test

Immediately after the scanning session, the participants completed a 1-min word reading test for each language (English-French participants: English and French lists; English-Chinese participants: English, Chinese, and French lists). Each list contained 160 high-frequency words. The English and French words had five to eight letters; the Chinese words had two characters. The words did not overlap with those in the main fMRI runs. Participants were instructed to read the words aloud not only as quickly as possible but also as clearly as possible. The order of languages was randomized for each participant. The number of words read within 1 min was recorded for each language (table S1).

A language dominance score was computed for each participant, based on their number of words read for the corresponding two languages tested in the main fMRI runs (abbreviated here as L and L′): [number of L words − number of L′ words] / [total number of L and L′ words]. The resulting scores corresponded well with the participants’ self-reported language history and dominance. The scores of the English-French participants fell along a continuum, with no clear boundaries between the early bilingual, English-dominant, and French-dominant subgroups (see fig. S5 and table S1). However, for English-Chinese participants, the dominance was always Chinese > English > French (Chinese-dominant).

### Data analysis

Data were analyzed in BrainVoyager v21.45 (Brain Innovation, Netherlands), MATLAB R2018b, and the MATLAB package NeuroElf v1.0 (https://neuroelf.net/).

#### Data preprocessing

The functional data underwent top-up distortion correction (COPE plugin in BrainVoyager), where the in-plane voxel displacement map between the first volumes of both the actual task run and its corresponding distortion correction run was computed, and applied to the task run. The distortion-corrected data were then corrected for slice scan time (sinc interpolation, slice scan order read from the slice time table in the DICOM headers), 3D rigid motion correction (trilinear for estimation, sinc for applying the correction, aligned to the first volume within each run), high-pass temporal filtering (GLM with Fourier basis set, number of cycles = 2). No spatial smoothing was applied to the data at this stage.

The MP2RAGE anatomical data consisted of four image types: inversion 1, inversion 2, quantitative T1, and uniform T1w. To obtain a similar appearance to the conventional MPRAGE anatomical data, the uniform image was divided by the T1 image (this step is optional), and the background noise was masked out by the inversion 2 image. The resulting anatomical image was resampled to 0.6 mm isotropic (framing cube dimensions: 384 × 384 × 384) and transformed into TAL space. For data visualization in figs. S2 and S6, the white matter–gray matter boundary was segmented in TAL space and reconstructed as surface meshes.

For fMRI across-run coregistration, we used a manual procedure and achieved better coregistration quality than the automatic procedure: The localizer run was coregistered to the anatomical data, and then all the other functional runs were manually coregistered to the localizer run. For quality checks, the cross-run coregistration quality was visually inspected with animations looping through the first volumes across the runs in TAL space. By coregistering with the same type of image modality (T2*), any mismatch between two runs (even in cases below 0.1 mm or 0.1°) became extremely easy to detect and correct. After the quality checks, all functional images were transformed into TAL space and were kept at 1.2-mm isotropic resolution.

#### Statistical analysis

All statistical tests in this study were two-tailed, including whole-brain contrasts.

##### Whole-brain GLM analysis

In the individual analysis, for both the localizer and the main fMRI runs, the blocks of conditions plus button presses for target trials were defined as predictors and were convolved with a canonical two-gamma hemodynamic response function (HRF); the six head-motion parameters were *z*-scored and defined as confound predictors. The target stimuli (stars and “######”) were not modeled, since they were collinear with the button presses. The time-course data were percent-transformed before running the fixed effects GLM, and the serial correlations in the data were corrected with an AR(2) model.

For the localizer, the group-level GLM was performed with the participants as the random effect, with both unsmoothed data and data smoothed with a 6-mm full width at half maximum of Gaussian filter. Whole-brain contrasts were initially thresholded at *P* < 0.001 and then underwent Monte-Carlo simulation for multiple-comparisons correction using the Cluster-level Statistical Threshold Estimator plugin in the BrainVoyager software: 5000 samples of null data with the same map smoothness as the real data were generated, and the contiguous cluster size that occurred in null data with an overall frequency lower than 0.05 (α < 0.05) was set as the cluster size threshold. For descriptive and visualization purposes (Fig. 2B), the event-related average time course per condition for the group-level cluster (and individual clusters/ROIs) was computed, subtracted, and divided by the baseline (activity from −2 to 0 TRs of each block).

Functional data smoothing was applied only to the group-level whole-brain analysis; no data smoothing was applied to subsequent individual-level analyses, either at the whole-brain level or at the individual ROI level.

##### Individual ROI definition

For each individual participant, the word- and face-specific clusters in the localizer were defined as ROIs (bilingual word contrast: average of English and French words > faces, bodies, houses, and tools; face contrast: faces > bodies, houses, tools, and average of English and French words; *P* < 0.001 uncorrected, trilinear interpolation, cluster size > 4). Two additional single language-specific contrasts (e.g., English > faces, bodies, houses, and tools) were computed for both groups to test whether the bilingual word-specific contrast included most of the language-specific voxels, which was the case for the English-French group but not for the English-Chinese group. Therefore, to maximize sensitivity for English-Chinese participants, the language-specific (Chinese-specific and English-specific) ROIs were defined by the main fMRI runs, which had many more block repetitions per condition than the English and Chinese word conditions in the localizer data (15 versus 5 repetitions). We used the positive and negative clusters from the contrast of average activity evoked by high- and low-frequency Chinese words versus activity evoked by English words, *P* < 0.001 uncorrected, trilinear interpolation, and a cluster size of >4. A similar analysis was attempted for French versus English words in English-French participants, but it yielded few clusters that were not reproducible across the localizer and main fMRI runs (see the section "Localizer for visual categories and overall reading circuit"). To avoid the possibility that we missed voxels sensitive only to English or French, in the Supplementary Materials, we computed contrasts for single languages (e.g., English words only > faces, bodies, houses, and tools), sorted voxels according to whether they overlapped with the bilingual word ROIs, and examined the single-condition activity profiles across languages within each kind of sorted voxels. The activity profiles were again inconsistent between the localizer and the main fMRI runs for English-French participants.

Throughout the study, the conditions used to examine ROI properties were independent of the conditions used to define the ROIs (conditions from different types of runs, i.e., the localizer versus the main fMRI runs, or different conditions within the same runs).

For each ROI, the broad anatomical region it belonged to (ventral occipitotemporal, lateral temporal, lateral frontal, IPS, medial frontal, and other) and the exact anatomical location were manually labeled according to (*81*) and the Duvernoy Human Brain Atlas. For VOTC ROIs, the subregions that they belonged to (EVC and fusiform) were further labeled, and the fusiform subregion was even further split into IOS-OTS, mFS, collateral sulcus, and middle temporal/occipital sulci/gyri according to individual anatomy. Clusters outside the brain, in the cerebrospinal fluid, or in the white matter were excluded as outliers. We also observed activation clusters having a shape of blood vessels (clearly visible in their 3D surface reconstructions). They overlapped or were adjacent to blood vessels, and yet some showed condition-specific activity with normal BOLD HRF shapes. The reason of their occurrence in 7-T fMRI data is not yet clear and could potentially be caused by the shift of B0 around venous blood vessels (*82*), a hypothesis that needs further investigation in future studies. In the current study, we excluded most of these vessel-shaped clusters and included only those with cluster sizes much larger than the vessels and having normal HRF shapes.

All ROIs after outlier exclusion were visualized as 3D surface meshes (see figs. S2 and S6). For each ROI, the averaged *X*, *Y*, and *Z* TAL coordinates across voxels were extracted for figure plotting and subsequent analyses. The single-condition β values (% signal change versus fixation) of the localizer and main-fMRI-run GLMs were also extracted per voxel and averaged per ROI. For both the English-French and English-Chinese groups, to examine the laterality of word- and face-specific clusters in the VOTC, the number of clusters and voxels were counted for each individual participant and subjected to a paired *t* test.

##### Word-similarity effects in ROIs

For English-French participants, we fitted the averaged β values for the 14 main conditions with the linear predictor of [1 2 2 3 4 5 5 6 7 8 8 9 10 10]/10 (normalized between 0 and 1 by dividing by 10), which assumes higher activity for higher frequencies within each word component (low English–low French frequency versus high English–high French frequency conditions within letters, bigrams, and quadrigrams) and higher activity for components more similar to words (letters < bigrams < quadrigrams < words) but assumes no difference in activity between languages (LE = LF, BE = BF, QE = QF, and WE = WF). The significance of the linear fit was corrected for multiple comparisons with FDR correction (BH procedure; https://www.mathworks.com/matlabcentral/fileexchange/27418-fdr_bh) across all participants and all ROIs or for ROIs within specific brain regions. The slope of the fit per ROI was used as a measure of the word-similarity effect for further analysis.

For English-Chinese participants, the linear predictor [1 2 3 4 5] (normalized between 0 and 1) was fitted separately to the first five English and Chinese conditions (avoiding the last two Chinese conditions, because they were used to define language-specific ROIs). No ROI survived the FDR correction, likely due to the much fewer conditions for fitting here compared to the English-French experiment (5 versus 14), rather than any quantitative differences in the data. We validated this possibility by fitting the five corresponding English conditions in the English-French participant data, resulting in a similar level of significance (0 of 773 ROIs survived FDR correction, compared to 499 of 773 ROIs when fitting all 14 conditions). Therefore, we have not reported FDR correction for the English-Chinese participants here. In addition, because there was no explicit correspondence of the word component levels between the English and Chinese conditions, we did not directly compare the English and Chinese slopes in the same participants.

##### Word and other functional gradients across VOTC word-specific ROIs

We examined the gradual posterior-to-anterior changes of several functions across VOTC ROIs (these changes across cortical spaces were termed gradients here). Within each individual participant across bilateral VOTC ROIs, one specific functional value per ROI was fitted to the TAL *Y* coordinates of the ROI, resulting in one regression coefficient per participant. A significant fit indicates a posterior-to-anterior change of that function. When examining the laterality effects of the gradients, the left- and right-hemispheric ROIs were fitted separately. The regression coefficients across participants then underwent a *t* test (one-sample *t* tests against 0 to examine the significance of the gradients and paired *t* tests between left and right hemispheres). For statistical tests involving the right-hemispheric ROIs, only participants with at least three right-hemispheric ROIs were included.

The functional gradients examined included (i) word-similarity slopes (separately for each hemisphere); (ii) activity evoked by English words, French words, false fonts, and numbers (across bilateral ROIs); (iii) the word selectivity index (separately for each hemisphere), computed as [word activity − other activity] / [word activity + other activity], where word activity is the averaged activity of English and French words and other activity is the averaged activity of faces, bodies, houses, and tools. We followed a padding procedure to ensure that the word selectivity index ranged from −1 to 1 (*83*): If the activity in the condition with the smallest amplitude was negative, all conditions were padded with the absolute activity value of that negative condition, so that the activity for all conditions was zero or positive.

(i) was computed using the main-fMRI-run data, for both the English-French and English-Chinese groups. A Kruskal-Wallis test was also performed to compare the English word gradients in word-specific ROIs between the two groups of participants. (ii) and (iii) served to further characterize word selectivity in VOTC, especially for ROIs in the anterior fusiform. They were computed with the localizer data and only for the English-French participants.

We also examined (i) for the lateral frontal ROIs in more detail. First, a PCA was performed on the TAL *Y* and *Z* coordinates across the bilateral frontal ROIs. The resulting first principal component was then used as the main direction of the putative frontal gradient and was correlated to the word-similarity slopes across ROIs.

##### Analyses of main-fMRI-run conditions in word-specific ROIs

Using GLMs and contrasts performed on the averaged time courses of each word-specific ROI, we examined the main-fMRI-run conditions in more detail, aiming to see if the potential lexicality effect, frequency effect, and language differences were associated with specific anatomical locations. See Figs. 1 and 5 for condition abbreviations.

For English-French participants, we examined the lexicality effect using the contrast [WE, WF > QE, QF]. Within each word component, we also examined the effect of component frequency, contrasting high-frequency conditions with low-frequency conditions in both languages (L+ > L−, B+ > B−, and Q+ > Q−). We further compared the difference of these frequency and lexicality effects between adjacent component levels ([B+ > B−] > [L+ > L−], [Q+ > Q−] > [B+ > B−], [WE, WF > QE, QF] > 2[Q+ > Q−]) to see if there was increased sensitivity for specific word components.

To examine the English-French language differences, we contrasted the conditions with high frequency in one language within each word component (LF > LE, BF > BE, and QF > QE). We also subjected the 2 × 2 data from each word component to a standard analysis of main effects and interactions. Taking the letter component as an example, the main effect of French was examined by the contrast [LF, L+ > LE, L−]; the main effect of English was examined by [LE, L+ > LF, L−]; and the interaction of French and English was examined by [L+ > LF] > [LE > L−].

For English-Chinese participants, for the English conditions, we performed the same contrasts for the frequency effect (L+ > L−, B+ > B−, and Q+ > Q−) and the contrast WE > Q+ for the lexicality effect. For the Chinese conditions, we contrasted adjacent conditions, including contrasts between noncharacter conditions (SG > S, RI > SG, and RP > RI), real characters > noncharacters (CP > RP), and contrasts involving real words (WL > CP and WH > WL).

##### Effect of individual language dominance

Separately within the English-French and English-Chinese groups, we examined whether the individual language dominance score (see the “One-minute word reading test” section) was correlated with brain activity differences between languages. Taking English and French as an example, the language dominance score was calculated as [number of English words − number of French words] / [total number of English and French words].

In the brains of individual participants, we first merged ROIs within each of the three broad regions (corresponding to the regions in Fig. 3 and described in detail in the “Individual ROI definition” section). Furthermore, we merged VOTC ROIs into subregions including EVC, fusiform, and even small subregions in the fusiform including the IOS-OTS subregion and the mFS subregion. We then averaged the single-condition β values within each region or subregion and computed the activity difference between English and French conditions (English-French group: localizer: WE − WF; main fMRI runs: LE − LF, BE − BF, QE − QF, WE − WF; English-Chinese group: WE − WC in both the localizer and the main fMRI runs, false fonts − Chinese strokes in the localizer). These activity differences for each region were then correlated to the language dominance scores across participants.

##### Nonparametric ICA analysis decomposing subvoxel components in word-specific ROIs

We used the code for nonparametric ICA analysis of (*35*) (https://github.com/snormanhaignere/nonparametric-ica), which maximizes the non-Gaussianity and finds independent components in the data. The English-French and English-Chinese data were analyzed separately. The input data were voxel β values for the 14 conditions in the main fMRI runs from all bilingual word-specific ROIs (those shown in Figs. 3 and 6, defined by the localizer). For English-Chinese participants, removing the overlapping voxels showing Chinese or English language specificity did not change the results. We searched for three components to allow us to separate the effects of the baseline, the word-similarity effect, and putative language differences. Searching for more components resulted in the same three stable components and some additional unstable components that were much harder to interpret. We report only the three stable components here.

This analysis resulted in three component profiles per voxel, each with a corresponding weight. The observed data could be reconstructed by multiplying the component profile and the weights. To derive the localizer activity profile corresponding to each component, we divided the observed localizer data by the main-fMRI-run component weights (Fig. 8).

## Supplementary Materials

### This PDF file includes:

Supplementary Text Figs. S1 to S9 Table S1 References

## Notes

View/request a protocol for this paper from *Bio-protocol*.
