# Supplementary material for: Does the visual word form area split in bilingual readers? A millimeter-scale 7-T fMRI study
Source: Sci Adv. 2023 Apr 5;9(14):eadf6140. doi: 10.1126/sciadv.adf6140 (PMC10075963; doi:10.1126/sciadv.adf6140)
Supplement: Supplementary file 1 — Supplementary Text Figs. S1 to S9 Table S1 References [file sciadv.adf6140_sm.pdf]

Science Advances
AAAS

Supplementary Materials for

Does the visual word form area split in bilingual readers? A millimeter-scale 7-T fMRI study

Minye Zhan *et al.*

Corresponding author: Minye Zhan, zhanminye@gmail.com

Sci. Adv. **9**, eadf6140 (2023)
DOI: 10.1126/sciadv.adf6140

This PDF file includes:

Supplementary Text
Figs. S1 to S9
Table S1
References

Supplementary Text

In the main text for the English-French participants, we analyzed the activity in word-specific clusters irrespective of language (hereafter referred to as “bilingual clusters”), defined by greater activity for the average of two languages (English and French) relative to other categories (faces, bodies, houses, tools) in the localizer runs ($p<0.001$ uncorrected, cluster size > 4). This was motivated by the finding that there were no consistent language-specific clusters when directly contrasting English and French words, neither in the localizer nor in the main fMRI runs, and that most of the language-specific voxels were included by the bilingual voxels (whole brain, English: 91.90%, French: 86.14%), especially in the VOTC (English: 95.75%, French: 94.82%). However, it may seem strange to pull together the two languages, and only then check whether they prefer one language over the other, and may miss language-specific clusters that didn’t survive thresholding under direct comparison.

In this supplementary analysis, we show that this choice of examining word-specific clusters without language specificity (bilingual clusters) in the English-French participants was inconsequential, because it did not lead to missing voxels that would be putatively strongly selective to one language. Here we performed two separate contrasts with each single language (English words > faces, bodies, houses, tools; French words > faces, bodies, houses, tools, $p<0.001$ uncorrected, cluster size > 4), separated all voxels into four categories, according to whether they overlapped with the bilingual clusters, and examined the averaged activity within each voxel category. If a language-specificity was present, it would be consistently observed in the language-specific conditions for word components (LE vs. LF, BE vs. BF, QE vs. QF) and for the real words (WE vs. WF). We performed this analysis in a VOTC mask (**Figure S3A**) and also in the remaining of the voxels outside the VOTC mask (**Figure S3B**). To minimize the possibility that potential language-dominance differences between participants may obscure the language-specificity activity differences, we performed the analysis separately for the three sub-groups. As a comparison, we also performed the same analysis for the English-Chinese participants.

For the English-French participants, both the group-averaged and the single-subject results showed that there was no consistent language-specificity for any of the three sub-groups in the single-language-specific voxels (**Figure S3** third and fourth columns). That is, the direction of language specificity for the word-components (LE vs. LF, BE vs. BF, QE vs. QF) did not match that of the real-word conditions, and the real-word conditions in the main fMRI runs were not even consistent with those of the localizer. Again, this supports our finding in the main text that the English-French participants use the same set of brain areas to process the written scripts of both English and French.

On the other hand, the language specificity of the English-Chinese participants was very consistent between the localizer and the main fMRI runs, regardless of whether they overlapped with the bilingual clusters or not. The single-language-specific non-overlapping voxels showed language specificity (higher activity amplitude), consistent with the finding that the bilingual word-specific voxels did not include all language-specific voxels. The slopes in the VOTC mask across conditions were also consistent with what we reported in the main text: In the bilingual clusters, a slope was present for both English and Chinese; however, in the Chinese-selective clusters though, a slope was present only for Chinese but not for English.

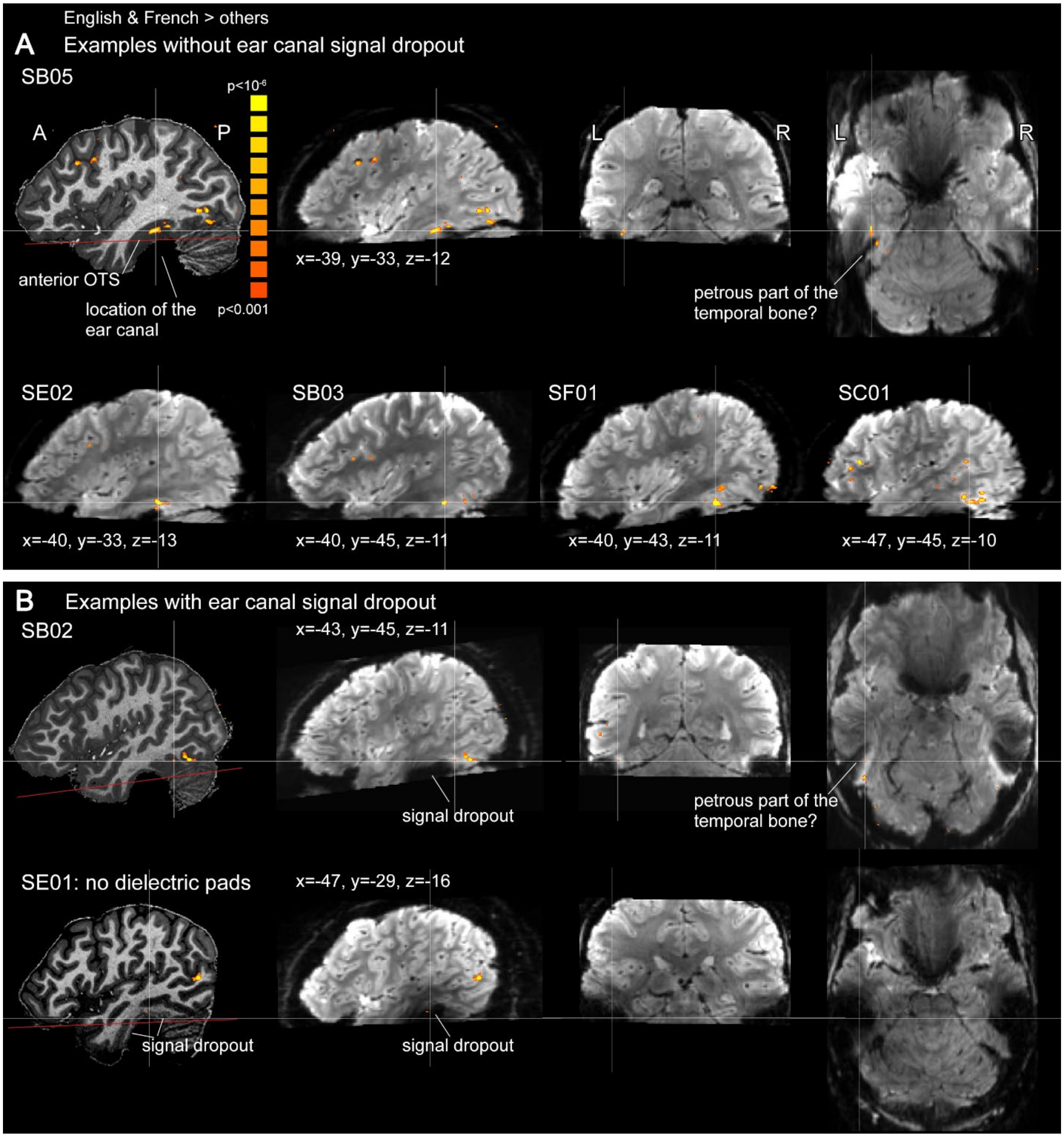

Figure S1. Functional data coverage and optimization for the ear canal signal dropout. The coverage for the majority of the datasets (28/31 across the two experiments) avoided the ear canal signal dropout. Panel **A** shows three orthogonal views from one participant (SB05), and sagittal views from one representative participant per group (SE02, SB03, SF01, SC01). Anterior VOTC word-specific activation clusters were observed in these participants. A minority of

datasets (3/31) were affected by signal dropout (SB01, SB02, SE01). Panel **B** shows the latter two participants. Note that even in this case, the clusters in the posterior VOTC (locations around the classic “VWFA”) were always present (TAL Y < -50, see **Figure S2**). The signal dropout was caused either by the inclusion of the ear canal into the data acquisition slab (SB01, SB02), or by not being able to use the dielectric pads in the tight space of the head coil (SE01). The resulting decrease in SNR in lateral temporal cortices can be observed in both the anatomical and functional images. In both A and B, the red lines overlaid on the anatomical images indicate the bottom edge of the data acquisition slabs, and were parallel to the in-plane phase encoding direction.

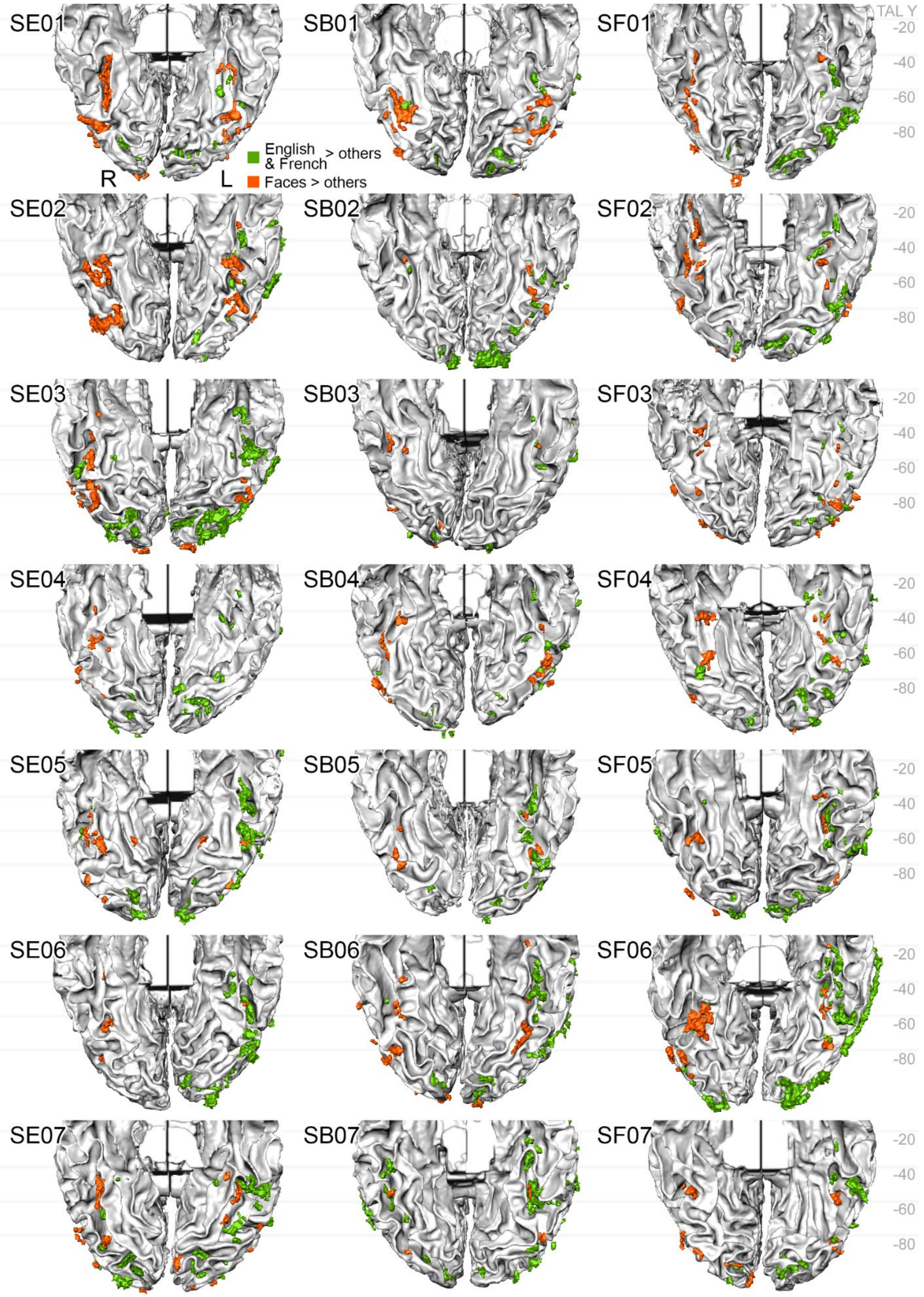

SE01
SB01
SF01
TAL Y
-20
-40
-60
-80
English & French > others
Faces > others
R
L
SE02
SB02
SF02
SE03
SB03
SF03
SE04
SB04
SF04
SE05
SB05
SF05
SE06
SB06
SF06
SE07
SB07
SF07

Figure S2. Word- and face-specific clusters in VOTC for each individual English-French participant ($p<0.001$ uncorrected). Word contrast: English & French words > faces, bodies, houses, tools; face contrast: faces > bodies, houses, tools, English & French words). The majority of participants (18/21) had anterior VOTC clusters (from TAL Y > -50 to the brainstem), except for the first 3 participants (SE01, SB01, SB02) affected by signal dropout above the ear canals.

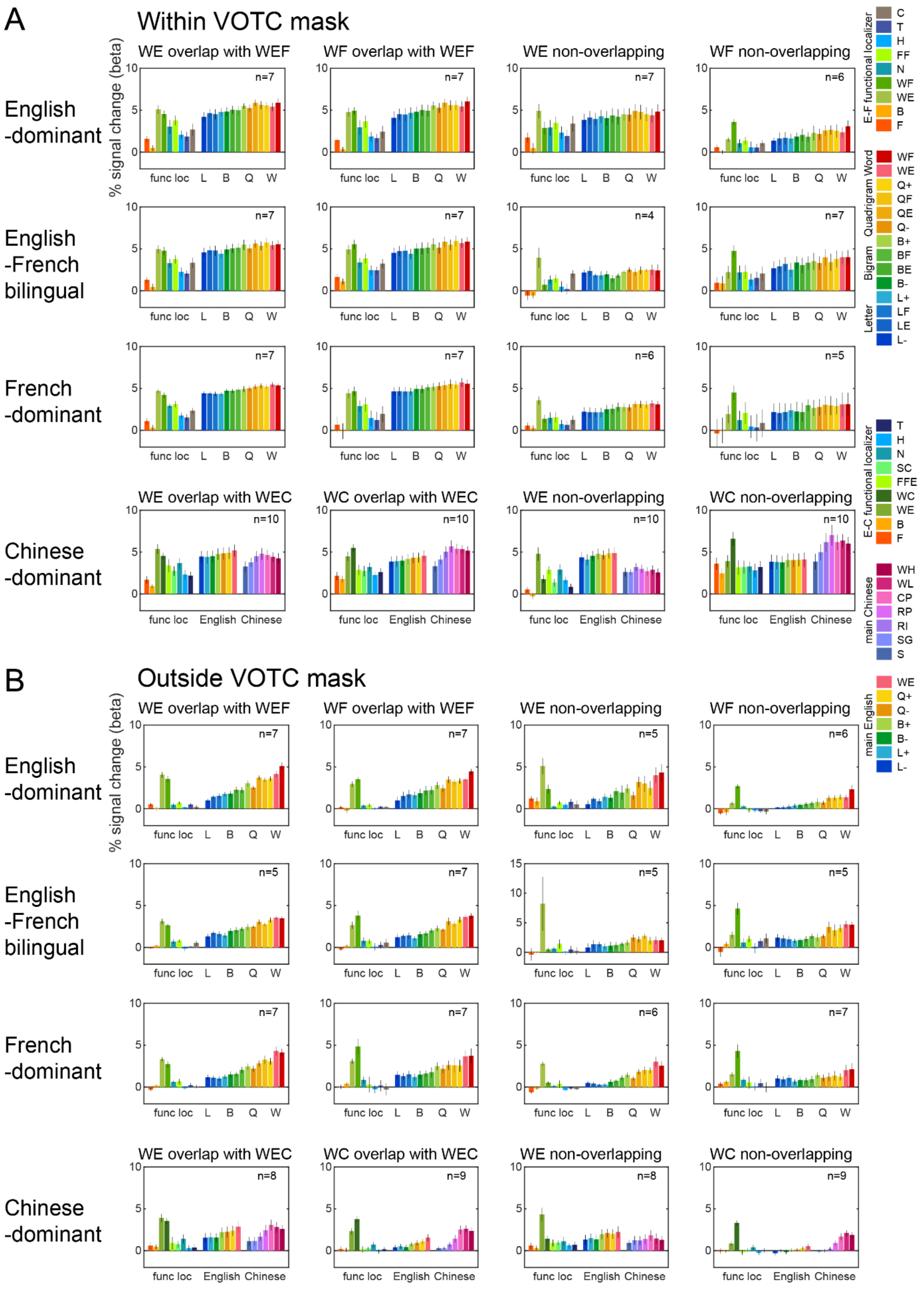
A
Within VOTC mask
WE overlap with WEF
WF overlap with WEF
WE non-overlapping
WF non-overlapping
English
-dominant
English
-French
bilingual
French
-dominant
% signal change (beta)
WE overlap with WEC
WC overlap with WEC
WC non-overlapping
Chinese
-dominant
func loc
L
B
Q
W
English
Chinese
E-F functional localizer
C
T
H
FF
N
WF
WE
B
F
Word
Quadrigram
Bigram
Letter
WF
WE
Q+
QF
QE
Q-
B+
BF
BE
B-
L+
LF
LE
L-
E-C functional localizer
T
H
N
SC
FFE
WC
WE
B
F
main Chinese
WH
WL
CP
RP
RI
SG
S
B
Outside VOTC mask
main English
WE
Q+
Q-
B+
B-
L+
L-

Figure S3. Average activity profiles for single-language-specific voxels that overlap or do not overlap with bilingual word-specific voxels. A. Voxels within the VOTC mask. **B.** Voxels outside the VOTC mask. For the English-Chinese (Chinese-dominant) participants, single-language-specific voxels not overlapping with bilingual word-specific voxels showed language specificity in the activity profiles, which was not the case for any sub-groups of English-French participants. The single-language-specific conditions are abbreviated as WE, WF, and WC. Error bars denote SEM across participants. The number of participants contributing to the average profiles is noted per plot.

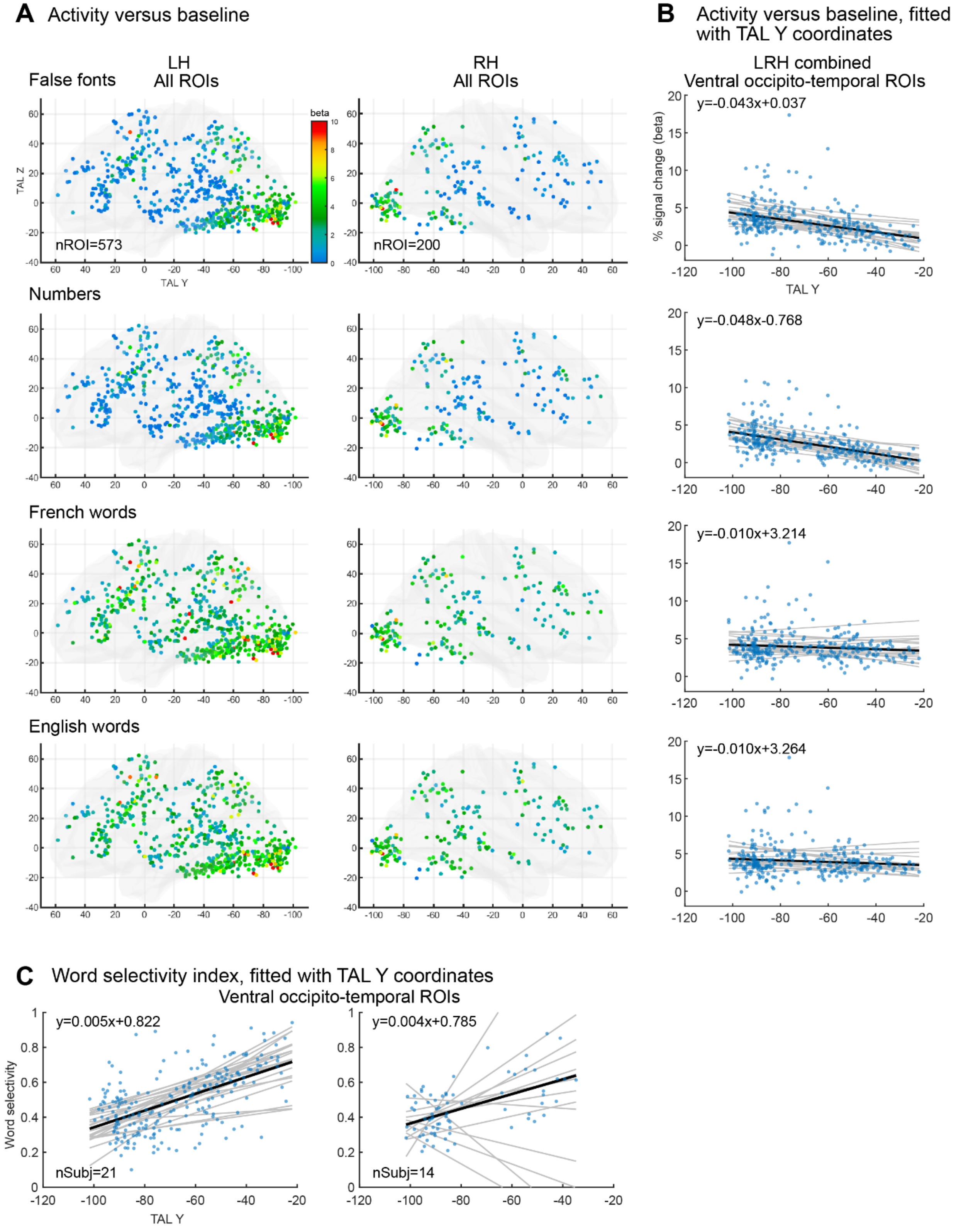
A Activity versus baseline
False fonts
LH
All ROIs
RH
All ROIs
nROI=573
nROI=200
beta
TAL Z
TAL Y
Numbers
French words
English words
B Activity versus baseline, fitted with TAL Y coordinates
LRH combined
Ventral occipito-temporal ROIs
% signal change (beta)
y=-0.043x+0.037
y=-0.048x-0.768
y=-0.010x+3.214
y=-0.010x+3.264
C Word selectivity index, fitted with TAL Y coordinates
Ventral occipito-temporal ROIs
Word selectivity
y=0.005x+0.822
y=0.004x+0.785
nSubj=21
nSubj=14

Figure S4. Gradient of word selectivity in word-specific ROIs across the cortex. **A.** Activity evoked by false fonts, numbers, French and English words, respectively, relative to the baseline in the localizer. All 4 conditions yielded similar activity in occipital and posterior ventral occipito-temporal ROIs, but there was a strong activity for words in anterior OTS. **B**. Response amplitudes in VOTC plotted against the TAL Y coordinates, corresponding to the conditions in **A** (ROIs combined across left and right hemispheres). **C**. Word selectivity indices in left and right ventral ROIs, plotted against TAL Y coordinates. The word selectivity index was computed as [word activity - other activity]/[word activity + other activity] (word activity: averaged activity of English and French words; other activity: averaged activity of faces, bodies, houses, tools). If a condition with the smallest activity amplitude was negative, all conditions were padded with that absolute value, so that the activity for all conditions was zero or positive, and the resulting word selectivity index would range from -1 to 1. In both **B** and **C**, the black lines are the linear fits to all ROIs across participants (parameters in the upper left corner), and the gray lines are the linear fits for individual participants.

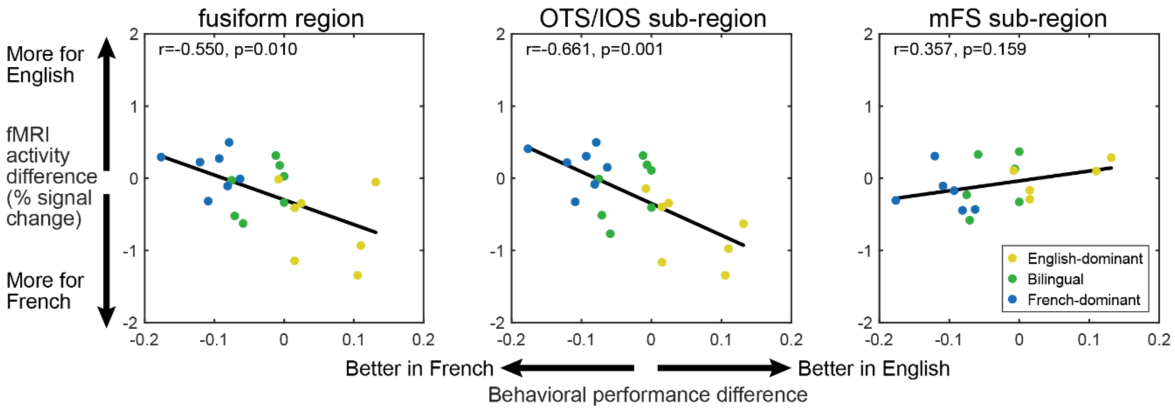

Figure S5. fMRI correlates of behavioral language dominance in the English-French bilingual readers. The difference in fMRI activity evoked in the fusiform region by English and by French words in the main fMRI runs (y axis) was negatively correlated with the difference in performance for those languages (x axis), indicating that less well mastered language yielded greater activity. The behavioral performance score was computed from the word counts in the one-minute reading task as [nWE - nWF] / [nWE + nWF], where nWE and nWF are the number of words read in English and French respectively.

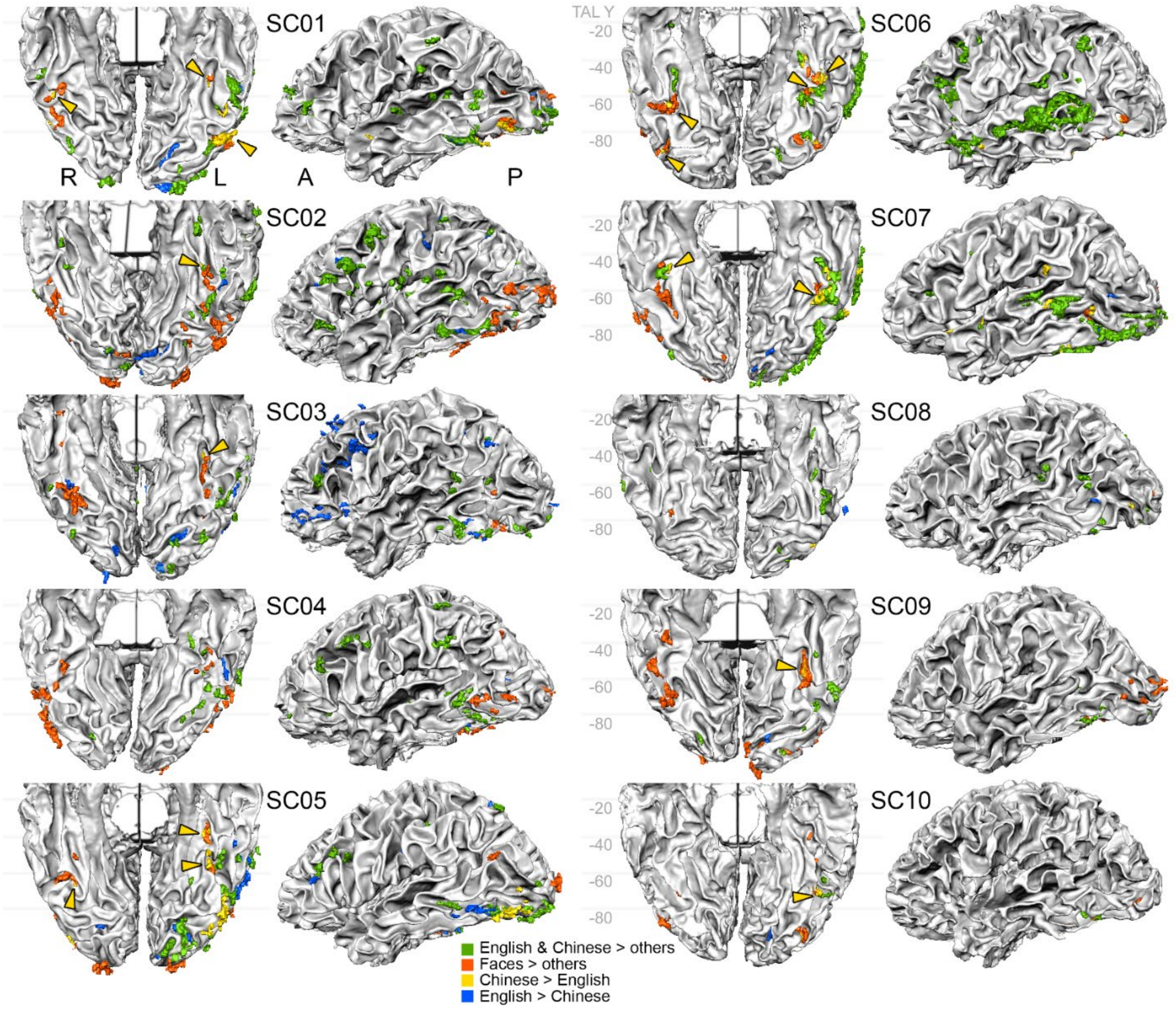

Figure S6. Word-, face-, and language-specific clusters in VOTC for each individual English-Chinese participant ($p<0.001$ uncorrected). The inferior and left lateral views of individual participants show four contrast maps. Two were from the localizer: English and Chinese words > faces, bodies, houses, tools; and faces > bodies, houses, tools, English and Chinese words. Two were from the main fMRI runs: Chinese words > English words and vice-versa. For better visibility, in the inferior view of the brains, Chinese-specific clusters localized close to face-specific clusters are marked with yellow arrows. 8/10 participants had such clusters (participant SC04 and SC08 lack them). For the English > Chinese contrast, participant SC03 was an outlier due to too many (more than 200) activated clusters at $p<0.001$, and the contrast was plotted at $p<0.0001$ instead.

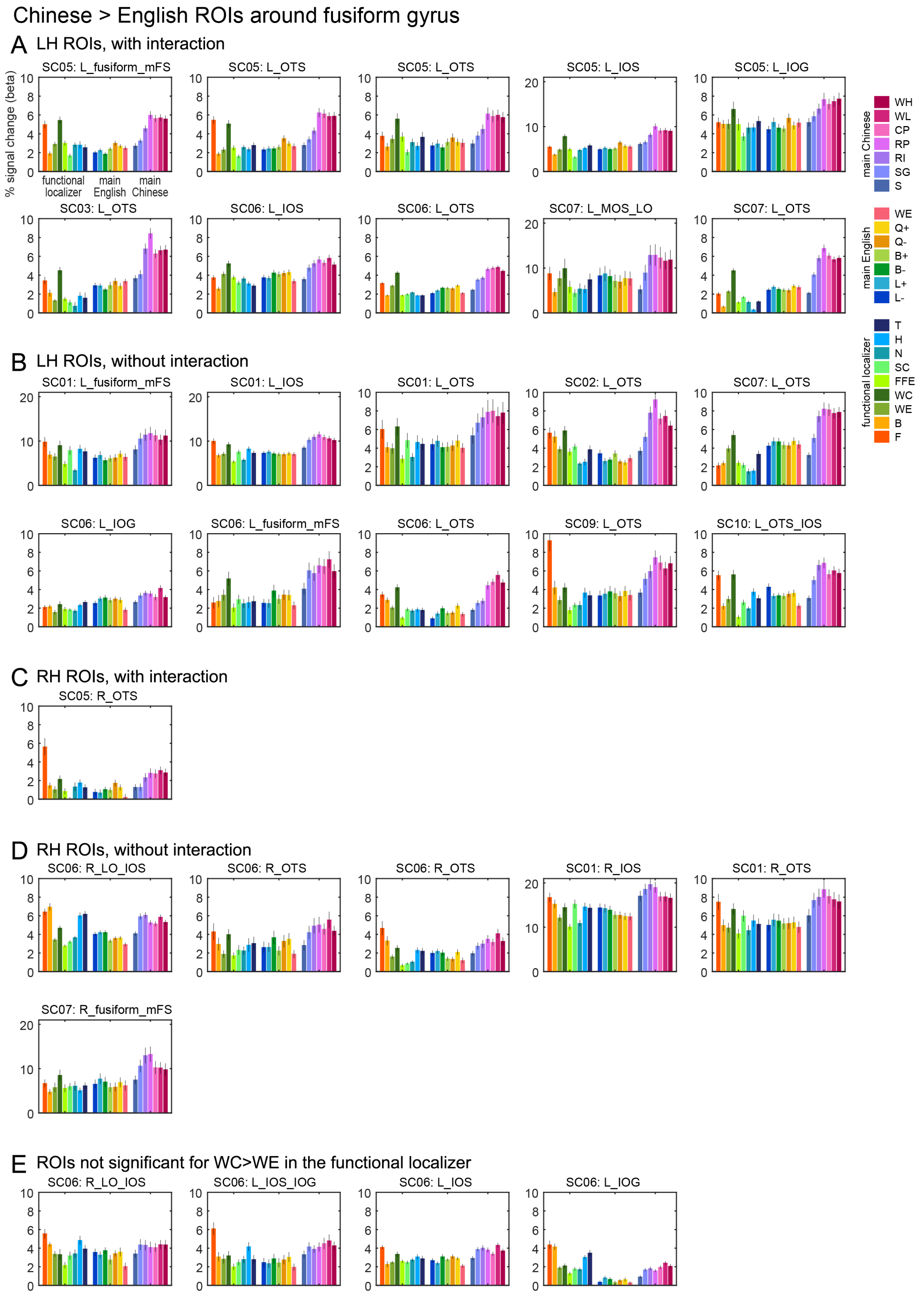

Chinese > English ROIs around fusiform gyrus
A LH ROIs, with interaction
SC05: L_fusiform_mFS
SC05: L_OTS
SC05: L_OTS
SC05: L_IOS
SC05: L_IOG
SC03: L_OTS
SC06: L_IOS
SC06: L_OTS
SC07: L_MOS_LO
SC07: L_OTS
% signal change (beta)
functional localizer
main English
main Chinese
B LH ROIs, without interaction
SC01: L_fusiform_mFS
SC01: L_IOS
SC01: L_OTS
SC02: L_OTS
SC07: L_OTS
SC06: L_IOG
SC06: L_fusiform_mFS
SC06: L_OTS
SC09: L_OTS
SC10: L_OTS_IOS
C RH ROIs, with interaction
SC05: R_OTS
D RH ROIs, without interaction
SC06: R_LO_IOS
SC06: R_OTS
SC06: R_OTS
SC01: R_IOS
SC01: R_OTS
SC07: R_fusiform_mFS
E ROIs not significant for WC>WE in the functional localizer
SC06: R_LO_IOS
SC06: L_IOS_IOG
SC06: L_IOS
SC06: L_IOG
main Chinese
WH
WL
CP
RP
RI
SG
S
main English
WE
Q+
Q-
B+
B-
L+
L-
functional localizer
T
H
N
SC
FFE
WC
WE
B
F

Figure S7. Activity profiles of all Chinese > English ROIs around the fusiform gyrus. ROIs are sorted by hemisphere and according to: first, whether the language-specific effect in the main fMRI runs was also significant in the localizer (**A-D**) or not (**E**); second, whether the interaction (WC – WE) > (SC – FFE) was significant, indicating that the Chinese > English difference was not present for retinotopically matched control stimuli, and thus was not purely driven by low-level visual features associated with each language. Participant ID and ROI names are indicated on each plot.

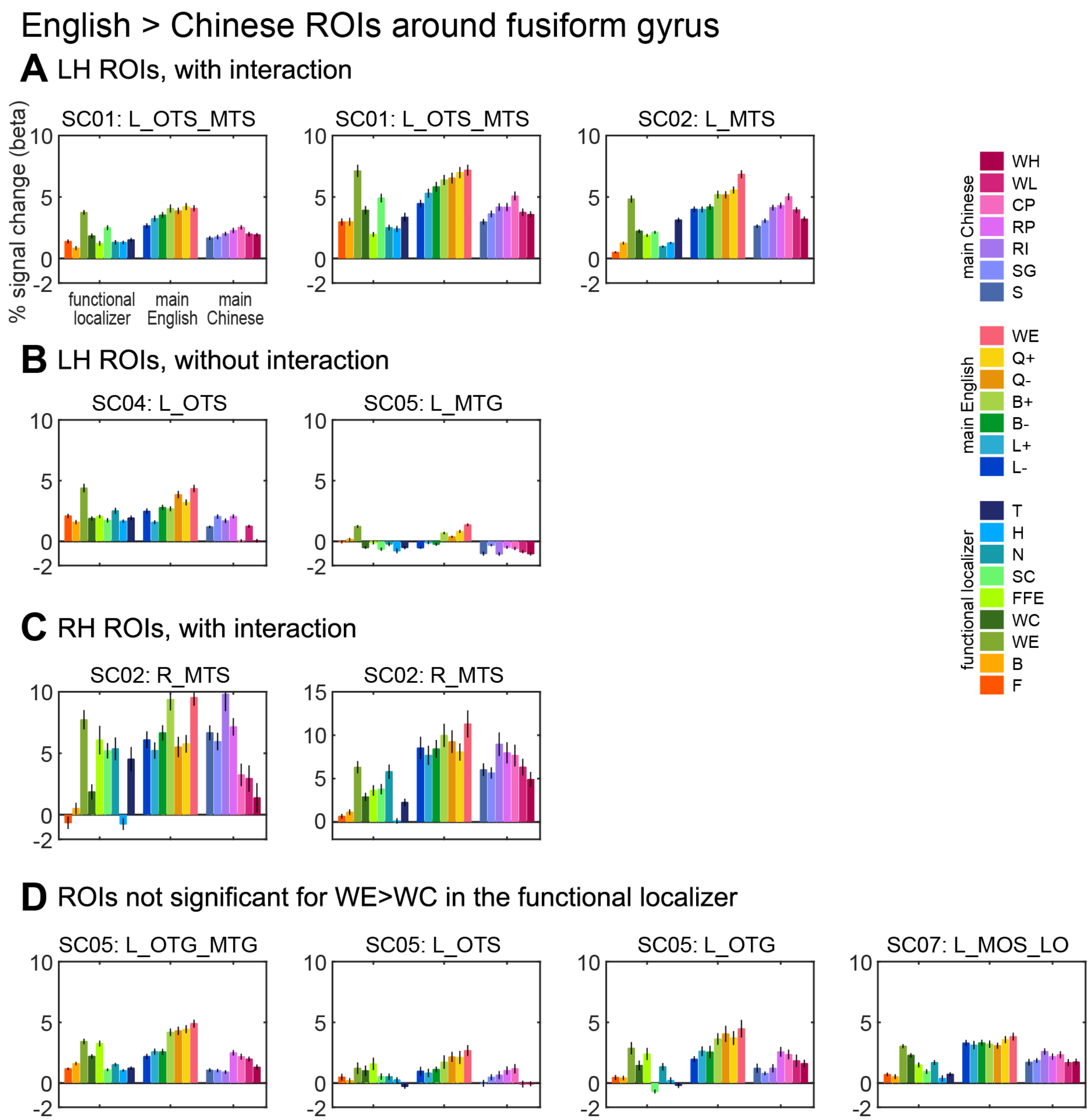

Figure S8. Activity profiles of all English > Chinese ROIs around the fusiform gyrus. ROIs are sorted in the same way as in **figure S7**.

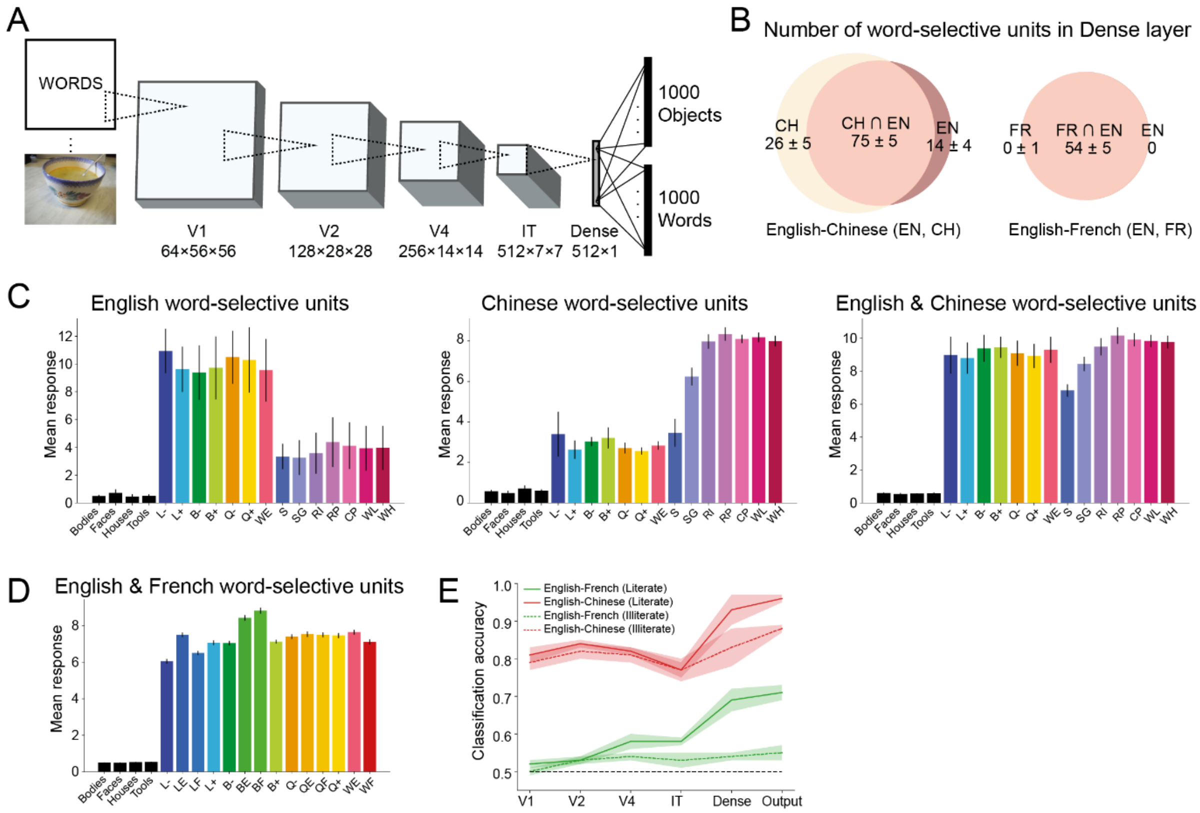

Figure S9. Simulating a bilingual reading network

A. In Hannagan et al. (*12*) we trained a biologically inspired convolutional neural network (CNN), Cornet-Z (*84*), to mimic a monolingual child's learning experience, an approach that we extend here to bilingualism. The CNN was initially trained (for 50 epochs) to classify a wide range of natural images from the ImageNet database (*85*). Once performance reached its ceiling on the image recognition task, the network was then simultaneously trained to recognize natural images and written words from two different languages, either English and French, or English and Chinese. To match the number of image categories, we chose 500 words from each language. Thus, the bilingual literate networks had 2000 classes, whereas the illiterate network had 1000 classes. For each word, we generated a large number of training examples that varied in their spatial location, size, font style, and case, similar to Hannagan et al. (*12*). The network achieved very high accuracy on words (86.73 ± 0.6 for English, and 96.94 ± 0.45 for Chinese in the English-Chinese network; and 88.86 ± 0.54 for English, and 88.3 ± 0.93 for French in the

English-French network), with only a minimal decrease in accuracy on the ImageNet pictures dataset. We trained a total of 5 instances of each network.

B. We labeled units in the dense layer as word-selective if their mean response to words from either language was more than 3 standard deviations above their mean response to natural image categories (i.e. bodies, faces, houses, and tools). Next, each of these word-selective units was labeled as language-selective if the difference between its mean response to the two languages was greater than 1 standard deviation. In the English-Chinese bilingual network, there were on average 114.2 (± 5.0) word-selective units, out of which 25.6 (± 4.8) were selective to the Chinese words and 13.8 (± 3.8) were selective to the English words. In contrast, in the English-French bilingual network, there were 53.8 (± 5.2) word-selective units, but none of them were language-selective.

C. Mean responses of the word-selective units to natural image categories (black bars) and to the main-fMRI-run stimuli from the English-Chinese experiment. The response averaged across word-selective units increased gradually for Chinese stimuli more similar to real characters, and showed a characteristic drop of response for the last 3 conditions, similar to what we observed in the English-Chinese participants (**Figure 7 and S7**). However, there was no word-similarity slope for the English stimuli, in agreement with the idea that this purely bottom-up network lacks the ability to simulate the word-similarity effects which are thought to primarily arise in a top-down manner (see ref. (*16*) and the general discussion in the main text). Error bars represent 1 standard deviation. See **Figure 5** for the bar legends.

D. Mean responses of the word-selective units to natural image categories (black bars) and to the main-fMRI-run stimuli from the English-French experiment. Again, only a hint of the word-similarity effect can be seen in this purely bottom-up network. See **Figure 1** for the bar legends.

E. Classification accuracy between the word stimuli from two languages across different bilingual and illiterate network layers. We used linear discriminant analysis to identify a linear combination of features that best classified the two languages. To avoid overfitting, we used 5-fold cross-validation. As expected, classification accuracy was higher for literate networks (solid

lines) than for the illiterate networks (dashed lines), and between Chinese and English stimuli (red lines) than between French and English stimuli (green lines), probably due to differences in low-level visual features. Shaded error bars represent 1 standard deviation.

Table S1. Individual language profiles. The age at which participants began acquiring the two languages was self-reported in the language-history questionnaire. Language dominance scores were derived from the number of words read in the one-minute word-reading test.

	Age of acquisition						Number of words read per minute			Language dominance score	
Participant	English spoken	English written	French spoken	French written	Chinese spoken	Chinese written	English	French	Chinese	English -French	Chinese-English
SE01	2	4	13	17	-	-	129	99	-	0.132	-
SE02	0	3	9	10	-	-	110	89	-	0.106	-
SE03	0	5	11	14	-	-	135	131	-	0.015	-
SE04	1	6	6	7	-	-	126	128	-	-0.008	-
SE05	3	4	3	10	-	-	125	119	-	0.025	-
SE06	0	5	13	13	-	-	134	130	-	0.015	-
SE07	0	3	9	9	-	-	136	109	-	0.110	-
SB01	1	5	1	5	-	-	96	108	-	-0.059	-
SB02	2	5	1	5	-	-	90	90	-	0.000	-
SB03	0	8	0	6	-	-	113	113	-	0.000	-
SB04	3	4	1	4	-	-	123	143	-	-0.075	-
SB05	0	10	0	5	-	-	118	136	-	-0.071	-
SB06	6	6	0	6	-	-	124	127	-	-0.012	-
SB07	1.5	4	1.5	4	-	-	157	159	-	-0.006	-
SF01	6	15	0	6	-	-	94	117	-	-0.109	-
SF02	8	8	0	5	-	-	141	160	-	-0.063	-
SF03	12	12	0	4	-	-	70	100	-	-0.176	-
SF04	8	8	0	5	-	-	136	160	-	-0.081	-
SF05	12	12	0	5	-	-	102	130	-	-0.121	-
SF06	8	8	0	4	-	-	112	135	-	-0.093	-
SF07	10	10	0	6	-	-	105	123	-	-0.079	-
SC01	4	5	23	23	1	5	97	80	119	0.096	0.102
SC02	3	7	18	18	2	3	99	83	123	0.088	0.108
SC03	4	10	18	18	1	6	86	63	102	0.154	0.085
SC04	9	10	17	18	1	4	79	65	114	0.097	0.181
SC05	12	12	21	22	0	6	106	95	123	0.055	0.074
SC06	6	12	19	20	1	3	110	98	137	0.058	0.109
SC07	4	6	18	18	0	2	59	55	160	0.035	0.461
SC08	10	17	19	24	0	9	121	114	134	0.030	0.051
SC09	11	14	22	24	0	9	93	87	100	0.033	0.036
SC10	6	18	22	24	0	5	50	47	62	0.031	0.107

REFERENCES AND NOTES

1. L. Cohen, S. Dehaene, L. Naccache, S. Lehericy, G. Dehaene-Lambertz, M. A. Henaff, F. Michel, The visual word form area: Spatial and temporal characterization of an initial stage of reading in normal subjects and posterior split-brain patients. *Brain* **123**, 291–307 (2000).

2. S. Brem, S. Bach, K. Kucian, T. K. Guttorm, E. Martin, H. Lyytinen, D. Brandeis, U. Richardson, Brain sensitivity to print emerges when children learn letter-speech sound correspondences. *Proc. Natl. Acad. Sci. U.S.A.* **107**, 7939–7944 (2010).

3. G. Dehaene-Lambertz, K. Monzalvo, S. Dehaene, The emergence of the visual word form: Longitudinal evolution of category-specific ventral visual areas during reading acquisition. *PLOS Biol.* **16**, e2004103 (2018).

4. S. Dehaene, F. Pegado, L. W. Braga, P. Ventura, G. Nunes Filho, A. Jobert, G. Dehaene-Lambertz, R. Kolinsky, J. Morais, L. Cohen, How learning to read changes the cortical networks for vision and language. *Science* **330**, 1359–1364 (2010).

5. C. I. Baker, J. Liu, L. L. Wald, K. K. Kwong, T. Benner, N. Kanwisher, Visual word processing and experiential origins of functional selectivity in human extrastriate cortex. *Proc. Natl. Acad. Sci. U.S.A.* **104**, 9087–9092 (2007).

6. M. Szwed, E. Qiao, A. Jobert, S. Dehaene, L. Cohen, Effects of literacy in early visual and occipitotemporal areas of Chinese and French readers. *J. Cogn. Neurosci.* **26**, 459–475 (2014).

7. U. Hasson, I. Levy, M. Behrmann, T. Hendler, R. Malach, Eccentricity bias as an organizing principle for human high-order object areas. *Neuron* **34**, 479–490 (2002).

8. B. Long, C.-P. Yu, T. Konkle, Mid-level visual features underlie the high-level categorical organization of the ventral stream. *Proc. Natl. Acad. Sci. U.S.A.* **115**, E9015–E9024 (2018).

9. F. Bouhali, M. Thiebaut de Schotten, P. Pinel, C. Poupon, J.-F. Mangin, S. Dehaene, L. Cohen, Anatomical connections of the visual word form area. *J. Neurosci.* **34**, 15402–15414 (2014).

10. Z. M. Saygin, D. E. Osher, E. S. Norton, D. A. Youssoufian, S. D. Beach, J. Feather, N. Gaab, J. D. E. Gabrieli, N. Kanwisher, Connectivity precedes function in the development of the visual word form area. *Nat. Neurosci.* **19**, 1250–1255 (2016).

11. R. Gaillard, L. Naccache, P. Pinel, S. Clemenceau, E. Volle, D. Hasboun, S. Dupont, M. Baulac, S. Dehaene, C. Adam, L. Cohen, Direct intracranial, FMRI, and lesion evidence for the causal role of left inferotemporal cortex in reading. *Neuron* **50**, 191–204 (2006).

12. T. Hannagan, A. Agrawal, L. Cohen, S. Dehaene, Emergence of a compositional neural code for written words: Recycling of a convolutional neural network for reading. *Proc. Natl. Acad. Sci. U.S.A.* **118**, e2104779118 (2021).

13. R. Rajalingham, K. Kar, S. Sanghavi, S. Dehaene, J. J. DiCarlo, The inferior temporal cortex is a potential cortical precursor of orthographic processing in untrained monkeys. *Nat. Commun.* **11**, 3886 (2020).

14. S. Dehaene, L. Cohen, M. Sigman, F. Vinckier, The neural code for written words: A proposal. *Trends Cogn. Sci.* **9**, 335–341 (2005).

15. F. Vinckier, S. Dehaene, A. Jobert, J. P. Dubus, M. Sigman, L. Cohen, Hierarchical coding of letter strings in the ventral stream: Dissecting the inner organization of the visual word-form system. *Neuron* **55**, 143–156 (2007).

16. O. Woolnough, C. Donos, P. S. Rollo, K. J. Forseth, Y. Lakretz, N. E. Crone, S. Fischer-Baum, S. Dehaene, N. Tandon, Spatiotemporal dynamics of orthographic and lexical processing in the ventral visual pathway. *Nat. Hum. Behav.* **5**, 389–398 (2021).

17. M. Xu, D. Baldauf, C. Q. Chang, R. Desimone, L. H. Tan, Distinct distributed patterns of neural activity are associated with two languages in the bilingual brain. *Sci. Adv.* **3**, e1603309 (2017).

18. J. Grainger, T. Dijkstra, On the representation and use of language information in bilinguals, in *Advances in Psychology* (Elsevier, 1992), vol. 83, pp. 207–220.

19. J. G. Rueckl, P. M. Paz-Alonso, P. J. Molfese, W.-J. Kuo, A. Bick, S. J. Frost, R. Hancock, D. H. Wu, W. E. Mencl, J. A. Dunabeitia, J.-R. Lee, M. Oliver, J. D. Zevin, F. Hoeft, M. Carreiras, O. J. L. Tzeng, K. R. Pugh, R. Frost, Universal brain signature of proficient reading: Evidence from four contrasting languages. *Proc. Natl. Acad. Sci. U.S.A.* **112**, 15510–15515 (2015).

20. H. Li, J. Zhang, G. Ding, Reading across writing systems: A meta-analysis of the neural correlates for first and second language reading. *Biling.: Lang. Cogn.* **24**, 537–548 (2021).

21. A. F. Wechsler, Dissociative alexia. *Arch. Neurol.* **34**, 257 (1977).

22. K. Srihasam, J. L. Vincent, M. S. Livingstone, Novel domain formation reveals proto-architecture in inferotemporal cortex. *Nat. Neurosci.* **17**, 1776–1783 (2014).

23. M. A. Changizi, Q. Zhang, H. Ye, S. Shimojo, The structures of letters and symbols throughout human history are selected to match those found in objects in natural scenes. *Am. Nat.* **167**, E117–E139 (2006).

24. Y. Sakurai, Kanji (morphogram) and Kana (phonogram) problem in Japanese alexia and agraphia, in *Frontiers of Neurology and Neuroscience*, J. Bogousslavsky, F. Boller, M. Iwata, Eds. (S. Karger AG, 2019), vol. 44, pp. 53–63.

25. T. N. Wydell, B. Butterworth, A case study of an English-Japanese bilingual with monolingual dyslexia. *Cognition* **70**, 273–305 (1999).

26. L. S. Glezer, M. Riesenhuber, Individual variability in location impacts orthographic selectivity in the "visual word form area". *J. Neurosci.* **33**, 11221–11226 (2013).

27. A. Puce, T. Allison, M. Asgari, J. C. Gore, G. McCarthy, Differential sensitivity of human visual cortex to faces, letterstrings, and textures: A functional magnetic resonance imaging study. *J. Neurosci.* **16**, 5205–5215 (1996).

28. N. Kanwisher, G. Yovel, The fusiform face area: A cortical region specialized for the perception of faces. *Phil. Trans. R. Soc. B.* **361**, 2109–2128 (2006).

29. D. Y. Tsao, S. Moeller, W. A. Freiwald, Comparing face patch systems in macaques and humans. *Proc. Natl. Acad. Sci. U.S.A.* **105**, 19514–19519 (2008).

30. A. Lochy, C. Jacques, L. Maillard, S. Colnat-Coulbois, B. Rossion, J. Jonas, Selective visual representation of letters and words in the left ventral occipito-temporal cortex with intracerebral recordings. *Proc. Natl. Acad. Sci. U.S.A.* **115**, E7595–E7604 (2018).

31. A. C. Nobre, T. Allison, G. McCarthy, Word recognition in the human inferior temporal lobe. *Nature* **372**, 260–263 (1994).

32. B. A. Wandell, The neurobiological basis of seeing words. *Ann. N. Y. Acad. Sci.* **1224**, 63–80 (2011).

33. S. Dehaene, A. Jobert, L. Naccache, P. Ciuciu, J.-B. Poline, D. Le Bihan, L. Cohen, Letter binding and invariant recognition of masked words: Behavioral and neuroimaging evidence. *Psychol. Sci.* **15**, 307–313 (2004).

34. W. A. Freiwald, D. Y. Tsao, Functional compartmentalization and viewpoint generalization within the macaque face-processing system. *Science* **330**, 845–851 (2010).

35. S. Norman-Haignere, N. G. Kanwisher, J. H. McDermott, Distinct cortical pathways for music and speech revealed by hypothesis-free voxel decomposition. *Neuron* **88**, 1281–1296 (2015).

36. S. V. Norman-Haignere, J. Feather, D. Boebinger, P. Brunner, A. Ritaccio, J. H. McDermott, G. Schalk, N. Kanwisher, A neural population selective for song in human auditory cortex. *Curr. Biol.* **32**, 1470–1484.e12 (2022).

37. M. J. Arcaro, T. Mautz, V. K. Berezovskii, M. S. Livingstone, Anatomical correlates of face patches in macaque inferotemporal cortex. *Proc. Natl. Acad. Sci. U.S.A.* **117**, 32667–32678 (2020).

38. L. Cohen, P. Salondy, C. Pallier, S. Dehaene, How does inattention affect written and spoken language processing? *Cortex* **138**, 212–227 (2021).

39. G. L. Krauss, R. Fisher, C. Plate, J. Hart, S. Uematsu, B. Gordon, R. P. Lesser, Cognitive effects of resecting basal temporal language areas. *Epilepsia* **37**, 476–483 (1996).

40. L. Schaffler, H. O. Luders, G. J. Beck, Quantitative comparison of language deficits produced by extraoperative electrical stimulation of Broca’s, Wernicke's, and basal temporal language areas. *Epilepsia* **37**, 463–475 (1996).

41. G. McCarthy, A. Puce, A. Belger, T. Allison, Electrophysiological studies of human face perception. II: Response properties of face-specific potentials generated in occipitotemporal cortex. *Cereb. Cortex* **9**, 431–444 (1999).

42. D. Y. Tsao, W. A. Freiwald, R. B. H. Tootell, M. S. Livingstone, A cortical region consisting entirely of face-selective cells. *Science* **311**, 670–674 (2006).

43. J. Liu, J. Tian, K. Lee, J. Li, A study on neural mechanism of face processing based on fMRI. *Prog. Nat. Sci.* **18**, 201–207 (2008).

44. J. Liu, J. Tian, J. Li, Q. Gong, K. Lee, Similarities in neural activations of face and Chinese character discrimination. *Neuroreport* **20**, 273–277 (2009).

45. J. J. Richler, I. Gauthier, A meta-analysis and review of holistic face processing. *Psychol. Bull.* **140**, 1281–1302 (2014).

46. J. Grainger, C. Whitney, Does the huamn mnid raed wrods as a wlohe? *Trends Cogn. Sci.* **8**, 58–59 (2004).

47. A. C.-N. Wong, C. M. Bukach, C. Yuen, L. Yang, S. Leung, E. Greenspon, Holistic processing of words modulated by reading experience. *PLOS ONE* **6**, e20753 (2011).

48. R. V.-y. Tso, R. T.-c Chan, Y.-f. Chan, D. Lin, Holistic processing of Chinese characters in college students with dyslexia. *Sci. Rep.* **11**, 1973 (2021).

49. P. Bao, L. She, M. McGill, D. Y. Tsao, A map of object space in primate inferotemporal cortex. *Nature* **583**, 103–108 (2020).

50. S. Dehaene, L. Cohen, Cultural recycling of cortical maps. *Neuron* **56**, 384–398 (2007).

51. X. Feng, K. Monzalvo, S. Dehaene, G. Dehaene-Lambertz, Evolution of reading and face circuits during the first three years of reading acquisition. *NeuroImage* **259**, 119394 (2022).

52. G. Golarai, A. Liberman, J. M. D. Yoon, K. Grill-Spector, Differential development of the ventral visual cortex extends through adolescence. *Front. Hum. Neurosci.* **3**, 80 (2010).

53. J. Gomez, M. A. Barnett, V. Natu, A. Mezer, N. Palomero-Gallagher, K. S. Weiner, K. Amunts, K. Zilles, K. Grill-Spector, Microstructural proliferation in human cortex is coupled with the development of face processing. *Science* **355**, 68–71 (2017).

54. W. J. B. van Heuven, T. Dijkstra, J. Grainger, Orthographic neighborhood effects in bilingual word recognition. *J. Mem. Lang.* **39**, 458–483 (1998).

55. N. I. Jamal, A. W. Piche, E. M. Napoliello, C. A. Perfetti, G. F. Eden, Neural basis of single-word reading in Spanish–English bilinguals. *Hum. Brain Mapp.* **33**, 235–245 (2012).

56. Y. N. Yum, P. J. Holcomb, J. Grainger, Words and pictures: An electrophysiological investigation of domain specific processing in native Chinese and English speakers. *Neuropsychologia* **49**, 1910–1922 (2011).

57. M. S. Koyama, J. F. Stein, C. J. Stoodley, P. C. Hansen, A cross-linguistic evaluation of script-specific effects on fMRI lateralization in late second language readers. *Front. Hum. Neurosci.* **8**, 249 (2014).

58. J. Qu, L. Zhang, C. Chen, P. Xie, H. Li, X. Liu, L. Mei, Cross-language pattern similarity in the bilateral fusiform cortex is associated with Reading proficiency in second language. *Neuroscience* (2019).

59. L. H. Tan, H. L. Liu, C. A. Perfetti, J. A. Spinks, P. T. Fox, J. H. Gao, The neural system underlying Chinese logograph reading. *Neuroimage* **13**, 836–846 (2001).

60. F. Cao, Neuroimaging studies of reading in bilinguals. *Biling.: Lang. Cogn.* **19**, 1–6 (2015).

61. C. A. Perfetti, Y. Liu, J. Fiez, J. Nelson, D. J. Bolger, L.-H. Tan, Reading in two writing systems: Accommodation and assimilation of the brain's reading network. *Biling.: Lang. Cogn.* **10**, 131–146 (2007).

62. S. Y. Kim, T. Qi, X. Feng, G. Ding, L. Liu, F. Cao, How does language distance between L1 and L2 affect the L2 brain network? An fMRI study of Korean-Chinese-English trilinguals. *NeuroImage* **129**, 25–39 (2016).

63. Y. Liu, S. Dunlap, J. Fiez, C. Perfetti, Evidence for neural accommodation to a writing system following learning. *Hum. Brain Mapp.* **28**, 1223–1234 (2007).

64. M. A. Changizi, S. Shimojo, Character complexity and redundancy in writing systems over human history. *Proc. Biol. Sci.* **272**, 267–275 (2005).

65. M. Amalric, S. Dehaene, Origins of the brain networks for advanced mathematics in expert mathematicians. *Proc. Natl. Acad. Sci. U.S.A.* **113**, 4909–4917 (2016).

66. J. Shum, D. Hermes, B. L. Foster, M. Dastjerdi, V. Rangarajan, J. Winawer, K. J. Miller, J. Parvizi, A brain area for visual numerals. *J. Neurosci.* **33**, 6709–6715 (2013).

67. L. Reich, M. Szwed, L. Cohen, A. Amedi, A ventral visual stream reading center independent of visual experience. *Curr. Biol.* **21**, 363–368 (2011).

68. E. Striem-Amit, L. Cohen, S. Dehaene, A. Amedi, Reading with sounds: Sensory substitution selectively activates the visual word form area in the blind. *Neuron* **76**, 640–652 (2012).

69. T. Hannagan, A. Amedi, L. Cohen, G. Dehaene-Lambertz, S. Dehaene, Origins of the specialization for letters and numbers in ventral occipitotemporal cortex. *Trends Cogn. Sci.* **19**, 374–382 (2015).

70. F. Bouhali, Z. Bezagu, S. Dehaene, L. Cohen, A mesial-to-lateral dissociation for orthographic processing in the visual cortex. *Proc. Natl. Acad. Sci. U.S.A.* **116**, 21936–21946 (2019).

71. W. Guo, S. Geng, M. Cao, J. Feng, Functional gradient of the fusiform cortex for Chinese character recognition. *eNeuro* **9**, 10.1523/ENEURO.0495-21.2022 (2022).

72. J. R. Binder, D. A. Medler, C. F. Westbury, E. Liebenthal, L. Buchanan, Tuning of the human left fusiform gyrus to sublexical orthographic structure. *Neuroimage* **33**, 739–748 (2006).

73. A. Agrawal, K. V. S. Hari, S. P. Arun, Reading increases the compositionality of visual word representations. *Psychol. Sci.* **30**, 1707–1723 (2019).

74. G. Hickok, D. Poeppel, The cortical organization of speech processing. *Nat. Rev. Neurosci.* **8**, 393–402 (2007).

75. C. J. Price, J. T. Devlin, The interactive account of ventral occipitotemporal contributions to reading. *Trends Cogn. Sci.* **15**, 246–253 (2011).

76. J. S. H. Taylor, K. Rastle, M. H. Davis, Can cognitive models explain brain activation during word and pseudoword reading? A meta-analysis of 36 neuroimaging studies. *Psychol. Bull.* **139**, 766–791 (2013).

77. V. Marian, H. K. Blumenfeld, M. Kaushanskaya, The language experience and proficiency questionnaire (LEAP-Q): Assessing language profiles in bilinguals and multilinguals. *J. Speech Lang. Hear. Res.* **50**, 940–967 (2007).

78. E. Keuleers, P. Lacey, K. Rastle, M. Brysbaert, The British lexicon project: Lexical decision data for 28,730 monosyllabic and disyllabic English words. *Behav. Res. Methods* **44**, 287–304 (2012).

79. B. New, C. Pallier, M. Brysbaert, L. Ferrand, Lexique 2: A new French lexical database. *Behav. Res. Methods Instrum. Comput.* **36**, 516–524 (2004).

80. Q. Cai, M. Brysbaert, SUBTLEX-CH: Chinese word and character frequencies based on film subtitles. *PLOS ONE* **5**, e10729 (2010).

81. B. Rossion, C. Jacques, J. Jonas, Mapping face categorization in the human ventral occipitotemporal cortex with direct neural intracranial recordings. *Ann. N. Y. Acad. Sci.* 10.1111/nyas.13596 (2018).

82. J. Winawer, H. Horiguchi, R. A. Sayres, K. Amano, B. A. Wandell, Mapping hV4 and ventral occipital cortex: The venous eclipse. *J. Vis.* **10**, 1 (2010).

83. W. K. Simmons, P. S. F. Bellgowan, A. Martin, Measuring selectivity in fMRI data. *Nat. Neurosci.* **10**, 4–5 (2007).

84. J. Kubilius, M. Schrimpf, A. Nayebi, D. Bear, D. L. K. Yamins, J. J. DiCarlo, CORnet: Modeling the neural mechanisms of Core object recognition. *Neuroscience* 10.1101/408385 (2018).

85. J. Deng, W. Dong, R. Socher, L.-J. Li, K. Li, L. Fei-Fei, ImageNet: A large-scale hierarchical image database, in *2009 IEEE Conference on Computer Vision and Pattern Recognition* (IEEE, 2009), pp. 248–255.
